# Widespread occurrence of covalent lysine–cysteine redox switches in proteins

**DOI:** 10.1038/s41589-021-00966-5

**Published:** 2022-02-14

**Authors:** Fabian Rabe von Pappenheim, Marie Wensien, Jin Ye, Jon Uranga, Iker Irisarri, Jan de Vries, Lisa-Marie Funk, Ricardo A. Mata, Kai Tittmann

**Affiliations:** 1 Department of Molecular Enzymology, Göttingen Center of Molecular Biosciences, Georg-August University Göttingen, Göttingen, Germany; 2 Department of Structural Dynamics, Max-Planck-Institute for Biophysical Chemistry, Göttingen, Germany; 3 Institute of Physical Chemistry, Georg-August University Göttingen, Göttingen, Germany; 4 Institute for Microbiology and Genetics & Göttingen Center of Molecular Biosciences, Georg-August University Göttingen, Göttingen, Germany; 5 Campus Institute Data Science, Georg-August University Göttingen, Göttingen, Germany

**Keywords:** X-ray crystallography, Enzymes, Post-translational modifications, Proteins

## Abstract

We recently reported the discovery of a lysine–cysteine redox switch in proteins with a covalent nitrogen–oxygen–sulfur (NOS) bridge. Here, a systematic survey of the whole protein structure database discloses that NOS bridges are ubiquitous redox switches in proteins of all domains of life and are found in diverse structural motifs and chemical variants. In several instances, lysines are observed in simultaneous linkage with two cysteines, forming a sulfur–oxygen–nitrogen–oxygen–sulfur (SONOS) bridge with a trivalent nitrogen, which constitutes an unusual native branching cross-link. In many proteins, the NOS switch contains a functionally essential lysine with direct roles in enzyme catalysis or binding of substrates, DNA or effectors, linking lysine chemistry and redox biology as a regulatory principle. NOS/SONOS switches are frequently found in proteins from human and plant pathogens, including severe acute respiratory syndrome coronavirus 2 (SARS-CoV-2), and also in many human proteins with established roles in gene expression, redox signaling and homeostasis in physiological and pathophysiological conditions.

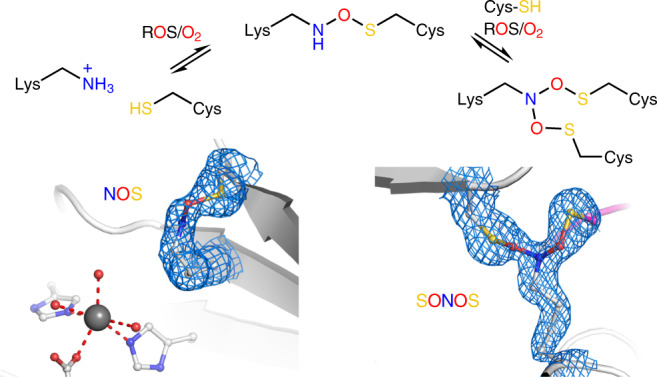

## Abstract (web summary)

A survey of protein structures identifies widespread lysine–cysteine cross-links in functionally diverse proteins across all domains of life and in various structural motifs, where these redox switches control enzyme catalysis and/or ligand binding.

## Main

**Fig. 1:**
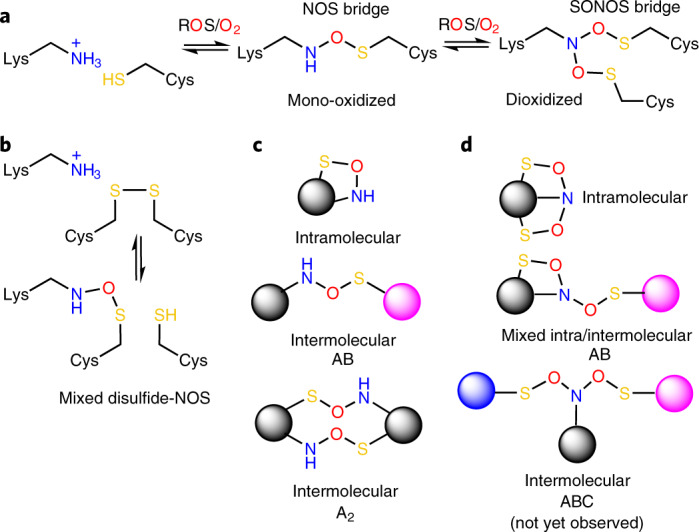
Chemical structures and topologies of NOS and SONOS redox switches in proteins. **a**, Structures and reaction scheme of NOS and SONOS redox bridge formation by ROS or oxygen in subsequent oxidation steps. **b**, Suggested structure of a ‘mixed’ NOS-disulfide redox switch, in which a disulfide is in equilibrium with an NOS bridge. **c**, Topologies of NOS bridges showing intramolecular and intermolecular cross-links as observed in experimentally determined protein structures. **d**, Topologies of SONOS bridges showing intramolecular and intermolecular cross-links as observed in experimentally determined protein structures. A SONOS bridge, where the lysine and two cysteines are contributed by three different proteins, has not been identified yet. The letters A, B and C indicate different proteins.

Reactive oxygen species (ROS) are central to redox signaling in all domains of life and critically control cell growth, development, metabolism, aging and the response to stress conditions, such as, for example, infection by pathogens^1–4^. At elevated levels, ROS induce oxidative stress that has been implicated in a myriad of pathologies, including cancer, neurodegenerative diseases, inflammation and autoimmune conditions^5,6^. The underlying molecular mechanisms of redox signaling and oxidative stress have been mostly attributed to chemical modifications of cysteine residues in redox-sensitive proteins with key reactions being disulfide bridge formation between two cysteines or oxidation, glutathionylation and nitrosylation of individual cysteines^7,8^. We have recently reported the discovery of an allosteric lysine–cysteine redox switch with a covalent NOS bridge that regulates the enzymatic activity in response to changing redox conditions (Fig. 1)^9^. In the redox-sensitive enzyme transaldolase from *Neisseria gonorrhoeae*, the side chains of neighboring residues Lys 8 and Cys 38 at the protein surface form an NOS cross-link under oxidizing conditions that leads to a loss of enzymatic activity through ‘structural crosstalk’ with the active site. Under reducing conditions, the cross-link is disengaged, and enzymatic activity is restored. It had remained unclear whether this lysine–cysteine cross-link is a widespread regulatory redox modification in proteins or a specific feature of the studied transaldolase protein^9^.

Here, we systematically mine the available protein structure database for proteins with undetected lysine–cysteine cross-links and find hitherto unidentified NOS bridges in proteins from all domains of life with critical roles in central cellular functions, including metabolism, gene expression, signaling, the ubiquitin pathway, DNA repair and redox homeostasis. These findings have wide-ranging biological implications in the context of redox signaling, oxidative stress and many human disease states.

## Results

### Geometric properties of NOS bridges

**Extended Data Fig. 1:**
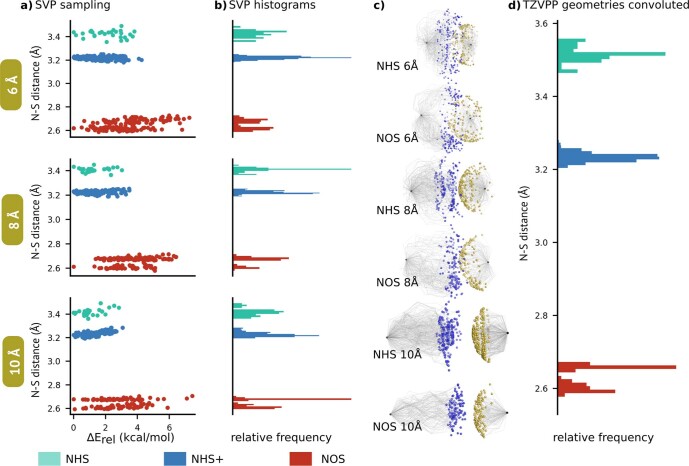
Computed structural information for conformers of Lys and Cys residues with and without the NOS bond. Computed structural information for conformers of Lys and Cys residues with and without the NOS bond, using different alpha-carbon distances (6 Å, 8 Å and 10 Å). The residues are computed as models, truncated at the alpha-carbons. The sampled structures at the semi-empirical level were refined at the B3LYP-D3(BJ)/def2-SVP level of theory (more details in the Supplementary Information), with the lowest conformers being reoptimised with a larger basis (def2-TZVPP). We have considered three different conformers: NOS - covalent bonding between the residues, NHS - hydrogen bond with the Lys deprotonated and NHS+ - hydrogen bond with the Lys protonated. (**a**) N-S distance (in Å) as a function of the relative conformer stability within each group computed with the SVP basis; (**b**) histogram for the SVP basis results; (**c**) overlap of SVP optimised structures for NHS and NOS; (**d**) histogram for all structures with the TZVPP basis. The images in **c** are obtained by an arbitrary alignment of the structures (given that only two points are fixed - the alpha-carbon positions). This can lead to somewhat different representations, or apparent ‘banding’ of the structures. This effect will depend on the alignment chosen and should not be misinterpreted. The figures are there to help illustrate the conformational freedom of these interactions..

Before we analyzed the deposited experimental protein structures in the protein database (www.rcsb.org) to search for potentially undetected covalent NOS bridges, we sought to define the geometric properties of covalent NOS bridges versus non-covalent hydrogen-bond interactions between cysteine and lysine residues to identify reliable criteria to discern between these scenarios. A survey of hydrogen-bond interactions of cysteines in protein structures disclosed mean interatomic S–N distances of 3.44 Å with lysine residues (cysteine as acceptor) and 3.75 Å when cysteine acts as a hydrogen-bond donor^10^. However, the closest possible physical encounter between the two side chains remained to be defined. To close this knowledge gap, we conducted quantum chemical calculations, where we analyzed the geometric properties of (1) the NOS bridge, (2) a hydrogen bond between lysine as amine (-NH_2_) and cysteine (-SH) and (3) a hydrogen bond between protonated lysine (-NH_3_^+^) and cysteine (-SH). An extensive conformational search was performed for the two residues in all three scenarios at different α-carbon atom distances (Extended Data Fig. 1). Several theoretical methods (also considering different dielectric constants; Supplementary Fig. 1) provided a consistent picture, whereby all minima featuring an N–S distance below 3.1 Å exhibited a covalent NOS bridge. The covalently bound NOS conformers displayed a bimodal distribution of the N–S 1,3 distance, with two maxima in the 2.6- to 2.7-Å range. This is a purely steric effect resulting from the relative positioning of the sulfur atom to the lysine chain (Supplementary Fig. 2). Hydrogen-bonded lysine–cysteine pairs are formed at much larger distances, with minimal values hovering at 3.2 Å for a protonated lysine–cysteine interaction, increasing to 3.5 Å once a proton is removed. The dipolar, charged Lys(NH_3_^+^)/Cys(S^–^) configuration was only obtained when applying a dielectric continuum with a dielectric constant *ε* > 10. Hereby, the N–S distances are slightly reduced but only down to 3.05 Å (Supplementary Fig. 3). This state was, however, less stable than the charge-neutral Lys(NH_2_)/Cys(SH) even up to dielectric constants mirroring water (within the limits of our model), leading to proton transfers. The computational results signal a clear difference between the various types of interactions, with the lowest energy distributions showing no overlap. The results are also consistent independent of the relative positioning of the two residues.

### Detectability of NOS bridges in protein crystal structures

**Extended Data Fig. 2:**
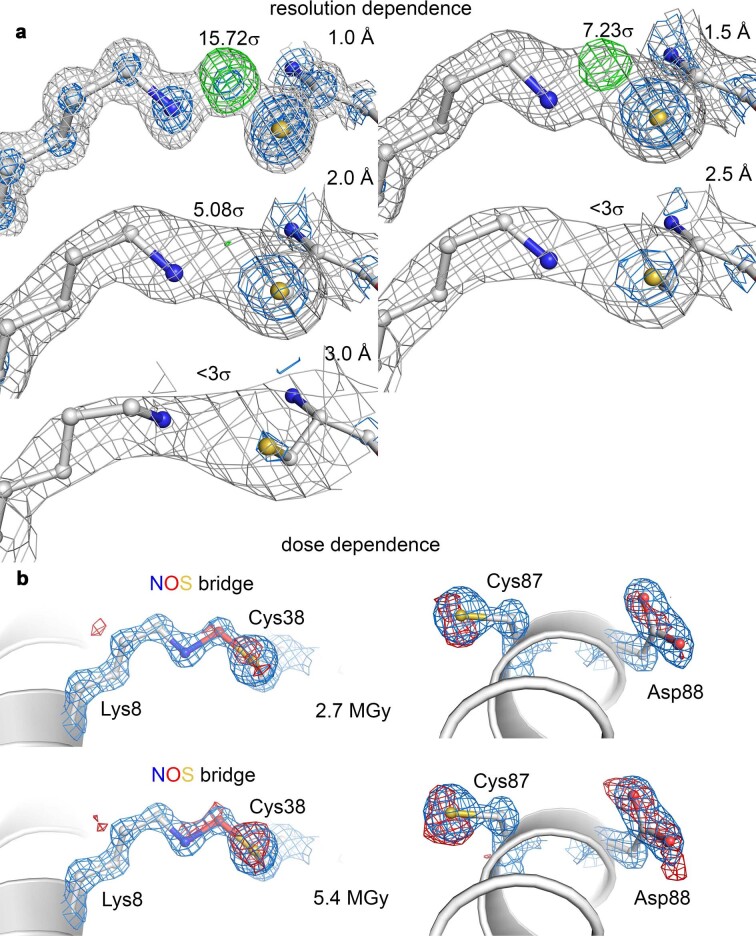
Detectability of NOS bridges in protein crystal structures in dependence from resolution and deposited dose. Detectability of NOS bridges in protein crystal structures in dependence from resolution and deposited dose. (**a**) Data truncation at different resolutions. A previously determined sub-ångstrøm resolution crystallographic dataset obtained for *Neisseria gonorrheae* transaldolase (pdb code 6XZ4)^9^, which forms an allosteric NOS bridge between Lys8 and Cys38, was truncated at different resolutions from 1.0-3.0 Å as indicated. For refinement, which included simulated annealing and B-factor blurring, the lysine and cysteine residues were modeled without the bridging oxygen atom. The calculated structural models and 2mFo-DFc electron density (blue: 3σ, grey: 1σ) and mFo-DFc difference electron density (green: 5σ) maps are shown. The respective intensities of the positive peaks in the difference electron density maps are indicated. Note the loss of structural information in both maps regarding the bridging oxygen atom with decreasing resolution. (**b**) Dose dependence. Crystallographic datasets for a single crystal of *Neisseria gonorrheae* transaldolase were collected at different doses 0.27, 2.7 MGy and 5.4 MGy at cryogenic temperature. The refined structural models of the NOS bridge formed between Lys8 and Cys38 and of a neighboring helix bearing residues Cys87 and Asp88 are shown for datasets obtained at doses of 2.7 MGy and 5.4 MGy. The respective 2mFo-DFc electron density maps shown in blue are contoured at 2σ. The calculated difference in intensity in the electron density maps relative to that obtained for a dose of 0.27 MGy is shown in red at a contour level of 3σ. Note the progressive loss in intensity for Cys87 and Asp88 with increasing dose, which indicates ‘radiation damage’. For the NOS bridge, radiation damage sets in at higher doses of 5.4 MGy.

Next, we tested the impact of resolution and X-ray dose in X-ray crystallographic experiments on the traceability of covalent NOS bridges in protein structures (Extended Data Fig. 2). We had initially detected the NOS redox switch in protein crystals that diffracted beyond 1 Å, a resolution that is rarely accomplished in protein crystallography but is required to unambiguously define the chemical nature of atoms and their connectivities^11^. We used a subångström dataset obtained for one of these crystals and truncated the diffraction data at 1.0, 1.5, 2.0, 2.5 and 3.0 Å and further omitted the bridging oxygen atom of the NOS bridge for structure refinements to eliminate model bias (Extended Data Fig. 2a). Maps were calculated with resolution-specific B-factor blurring and simulated annealing of the NOS bridge lysine and cysteine residues. While a data truncation is physically not the same as obtaining datasets at different resolutions, this approach illustrates the general trends. As one would expect, the detectability of the covalent cross-link is critically dependent on the resolution: while the presence of the bridging atom is verifiable up to resolutions of 2 Å based on both the 2*mF*_o_*–DF*_c_ as well as *mF*_o_–*DF*_c_ difference electron density maps (positive difference peaks), the interpretation of electron density maps at lower resolutions becomes ambiguous. In the transaldolase protein crystals we analyzed, the NOS bridge exhibits almost full occupancy, and our system may therefore be regarded as a best-case scenario (also considering the low B-factors). To test the impact of high-energy synchrotron radiation on the NOS bridge in protein crystals, which is known to promote photochemical reactions of redox-sensitive groups^12^, we conducted dose-dependent experiments depositing doses between 0.27 and 5.4 MGy in 0.27-MGy increments (Extended Data Fig. 2b; X-ray crystallographic statistics in Supplementary Table 1). These experiments reveal that the NOS bridge in the tested protein crystals is relatively radiation hard and withstands doses up to 2.7 MGy, where radiation damage at neighboring acidic side chains becomes visible. When we deposited high doses of 5.4 MGy, we detected a slight decomposition of the NOS bridge. In sum, to reliably detect NOS bridges in protein crystal structures, a resolution of better than 2 Å is required as well as a high occupancy of this group and sufficient local order. Photochemistry during data collection is unlikely to cause NOS bridge formation artificially, as we have discussed before^9^; to the contrary, NOS bridges decompose while interacting with high-energy radiation but only at doses far higher than commonly used for data collection^12^.

**Extended Data Fig. 3:**
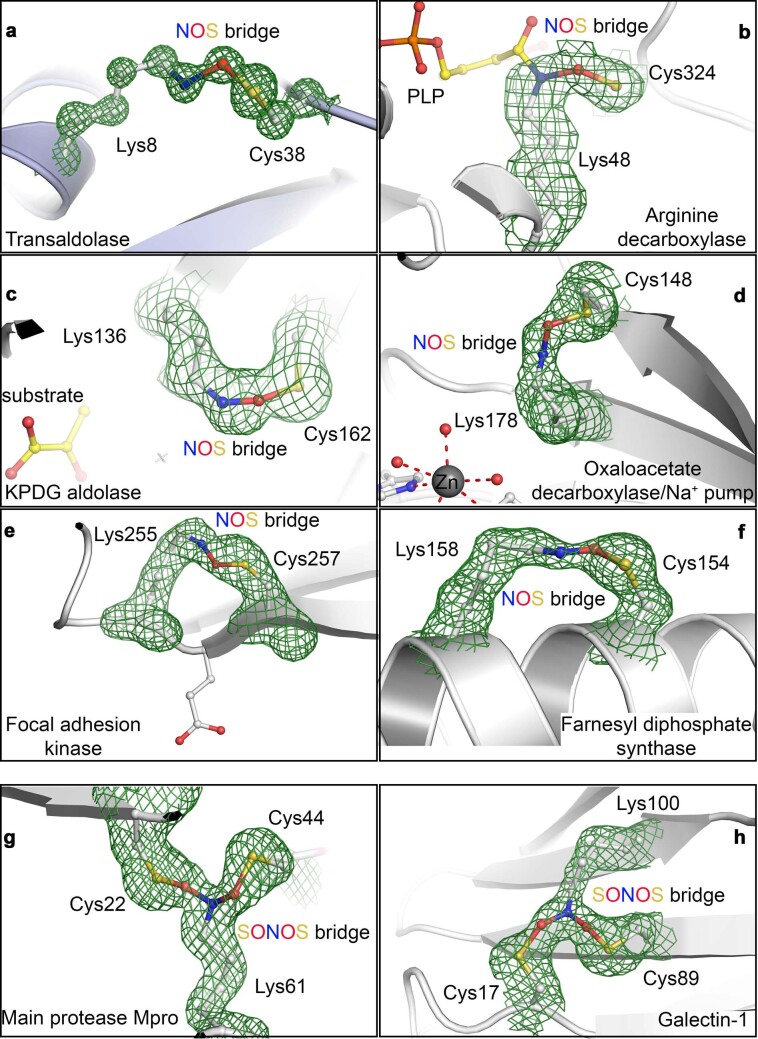
Unbiased mFo-DFc omit maps for NOS and SONOS bridges in representative examples calculated by Phenix.polder^59^. Unbiased mFo-DFc omit maps for NOS and SONOS bridges in representative examples calculated by Phenix.polder^59^. The lysine and cysteine residues incl. the NOS or SONOS bridges were excluded from the structural models prior to map calculation to eliminate model bias. The corresponding pdb codes and contour levels are indicated. (**a**) transaldolase from *Neisseria gonorrhoeae* (pdb code 6ZX4, 5σ), (**b**) arginine decarboxylase from *Paramecium bursaria* Chlorella virus (pdb code 2NV9, 3.5σ), (**c**) KPDG aldolase from *Oleispira antarctica* (pdb code 3VCR, 4σ), (**d**) oxaloacetate decarboxylase/Na^+^ pump from *Vibrio cholerae* (pdb code 2NX9, 3σ), (**e**) focal adhesion kinase from *Gallus gallus* (pdb code 6CB0, 5σ), (**f**) farnesyl diphoshate synthase from *Trypanosoma cruzi* (pdb code 6SDP, 3σ), (**g**) main protease from SARS-CoV-2 (pdb code 7JR4, 5σ) and (**h**) galectin-1 from *Rattus norvegicus* (pdb code 4GA9, 3σ).

**Extended Data Fig. 4:**
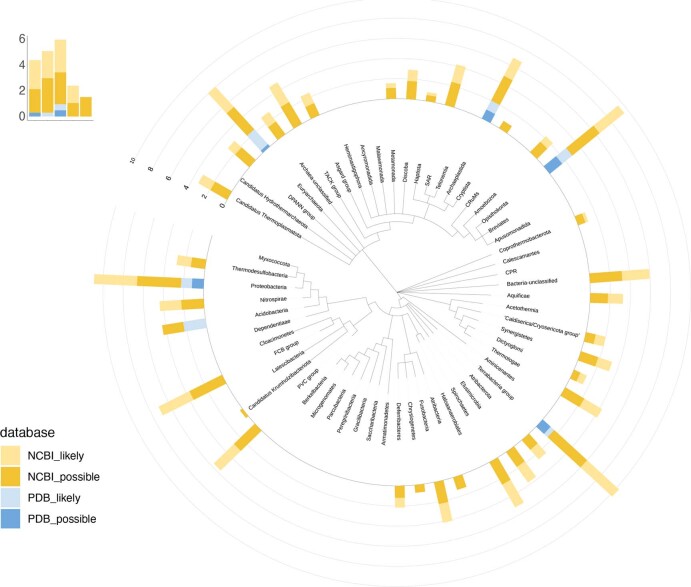
Distribution of proteins containing NOS crosslinks across the Tree of Life. Distribution of proteins containing NOS crosslinks across the Tree of Life. Shown are proteins likely/ probably containing NOS bridges (blue) as identified from PDB structures (Supplementary Data 1), as well as homologs of these proteins as identified in NCBI’s non-redundant (NR) database (yellow). Values are log10-transformed.

On the basis of the quantum chemical calculations and the X-ray crystallographic experiments on our model system, we downloaded all Protein Data Bank (PDB) entries with a resolution of ≤2 Å (65,000). We then searched for close contacts between lysine and cysteine residues, specifically pairs with interatomic N–S distances of less than 3 Å, which are in the regimen of covalent NOS bridges rather than hydrogen-bond interactions (>3.2 Å). We identified 266 deposited structures with ~400 potential NOS bridges (Supplementary Data 1) and manually inspected the putative lysine–cysteine cross-link site in all of the structures, for which structure factors and electron density maps were available. To eliminate a potential model bias, we also calculated *mF*_o_–*DF*_c_ omit difference electron density maps, where the NOS bridge residues had been excluded from the structural models (representative examples are shown in Extended Data Fig. 3). The corresponding lysine and cysteine residues were modeled without a cross-link in almost all deposited structures, with a methylene bridge in very few structures and with an NOS link in one structure except our own structures. We remodeled both residues with a covalent cross-link either as an NOS bridge or, alternatively, as a direct N–S linkage (sulfenamide) using geometrically parametrized restraints obtained by our quantum chemical calculations. We consider formation of a methylene (N–CH_2_–S) bridge as highly unlikely, as previously discussed by us and others^9,13^. We then re-refined the structures and compared the obtained models and electron density maps for the three alternative scenarios: (1) a covalent NOS linkage, (2) a covalent N–S linkage and (3) a non-covalent hydrogen-bond interaction (as deposited). In ~150 datasets, the existence of an NOS bridge is very likely (~100) or possible (~50), while a non-covalent hydrogen-bonding interaction or direct N–S linkage can be relegated in these datasets to a minor probability (Supplementary Data 1). In a few datasets, the electron density maps clearly indicate the presence of a cross-link, but the quality is insufficient to discriminate between an NOS bridge and a direct N–S bond (sulfenamide; Supplementary Data 1). Proteins likely or possibly containing NOS cross-links and corresponding homologs are found in all domains of life (Extended Data Fig. 4). Proteins from human or plant pathogens (Supplementary Table 2) and human proteins (Supplementary Table 3) are summarized individually.

### Structural and chemical motifs of NOS bridges

**Fig. 2:**
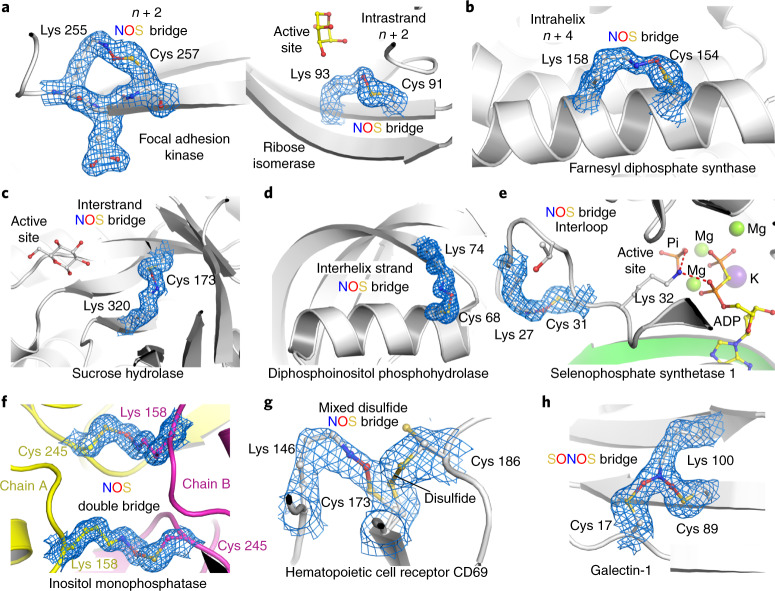
Structural and chemical motifs of NOS bridges in proteins. The corresponding 2*mF*_o_–*DF*_c_ electron density maps are shown in blue at a contour level of 1*σ*. **a**, Examples for NOS bridges in intrastrand or strand-like motifs with a sequence distance of *n* + 2. Shown are focal adhesion kinase from *Gallus gallus* (PDB: 6CB0) and ribose isomerase from *Acinetobacter* sp. (PDB: 4Q0P). **b**, Example for an NOS bridge in intrahelix motifs with a sequence distance of *n* + 4. Shown is the farnesyl diphosphate synthase from *Trypanosoma cruzi* (PDB: 6SDP). **c**, Example for an NOS bridge in interstrand (cross-strand) motifs. Shown is the sucrose hydrolase from *Xanthomonas axonopodis* (PDB: 3CZG). **d**, Example for an NOS bridge connecting a helix and a neighboring strand showing human diphosphoinositol phosphohydrolase (PDB: 6PCK). **e**, Example for an intraloop NOS bridge showing human selenophosphate synthetase 1 (PDB: 3FD5). **f**, Example for an intermolecular NOS double bridge between two chains in a homodimeric assembly. Shown is the inositol monophosphatase from *Medicago truncatula* (PDB: 5EQA). The two chains are colored individually in yellow and magenta, respectively. **g**, Example for a ‘mixed’ NOS-disulfide switch showing the human hematopoietic cell receptor CD69 (PDB: 1E8I, chain A) with an NOS bridge between Lys 146 and Cys 173 (30% occupancy) and a disulfide bridge between Cys 173 and Cys 186 (70% occupancy). **h**, Example for a SONOS bridge linking a lysine and two cysteines at the same time showing galectin-1 from *Rattus norvegicus* (PDB: 4GA9).

**Fig. 3:**
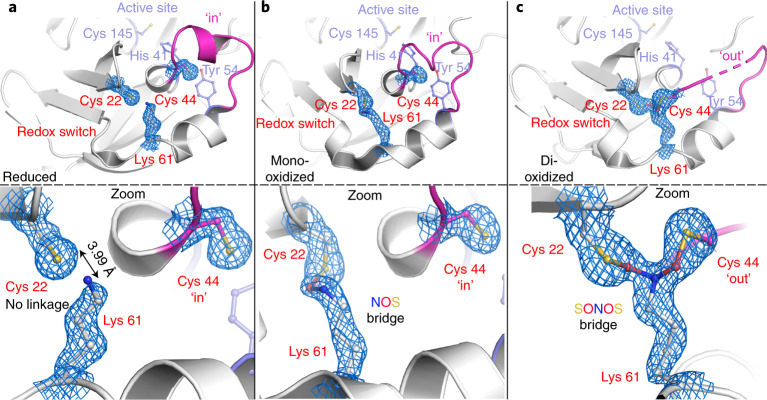
NOS and SONOS bridges in Mpro from SARS-CoV-2. The corresponding 2*mF*_o_–*DF*_c_ electron density maps are shown in blue at a contour level of 1*σ*. **a**, Structure of Mpro in the reduced state (PDB: 7JR3) showing the redox switch at the protein surface formed by residues Cys 22, Cys 44 and Lys 61 (highlighted in red) and the active site with residues Cys 145 (catalytic nucleophile), His 41 and Tyr 54 (highlighted in slate blue). A mobile loop bearing Cys 44 is indicated in magenta. Top, structural overview; bottom, close-up of the redox switch site. Note that residue Cys 44 is in the ‘in conformation’ and interacts with Tyr 54. **b**, Structure of Mpro in a mono-oxidized state with an NOS bridge formed between Cys 22 and Lys 61 (PDB: 6XMK). Top, structural overview; bottom, close-up of the redox switch site. Cys 44 is found in the ‘in conformation’. **c**, Structure of Mpro in a dioxidized state with a SONOS bridge formed between Cys 22, Lys 61 and Cys 44 (PDB: 7JR4). Top, structural overview; bottom, close-up of the redox switch site. Cys 44 is found in the ‘out conformation’. Competitive refinements (SONOS bridge only, two separate NOS bridges, mixture of SONOS and NOS) indicate full occupancy of the SONOS bridge.

**Extended Data Fig. 5:**
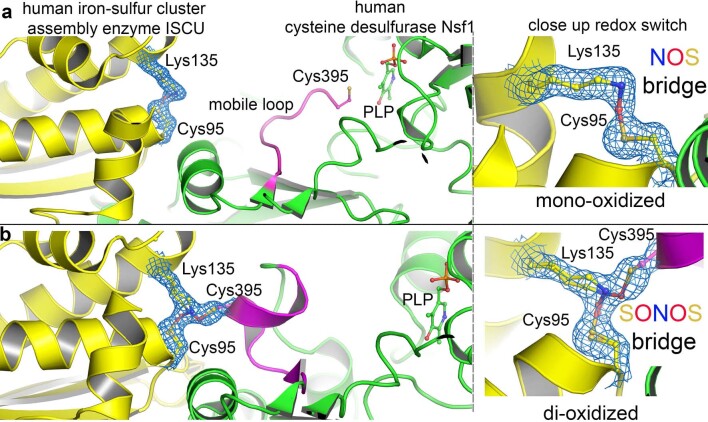
NOS and SONOS bridges in the human Fe/S cluster biosynthesis complex. NOS and SONOS bridges in the human Fe/S cluster biosynthesis complex. The corresponding 2mFo-DFc electron density maps are shown in blue at a contour level of 1σ. (**a**) Structure of the complex consisting of iron-sulfur cluster assembly enzyme ISCU (highlighted in yellow) and cysteine desulfurase Nsf1 (highlighted in green) in the mono-oxidized state with an intramolecular NOS bridge between Cys95 and Lys131 of ISCU (pdb code 6UXE). A mobile loop from Nsf1 with catalytic residue Cys395 catalyzing sulfide transfer from the PLP cofactor at the active site of Nsf1 to ISCU is indicated in magenta. Left panel: structural overview. Right panel: close-up of the redox switch site. Note that Cys395 visits the active site of Nsf1. (**b**) Structure of the complex in the di-oxidized state with a SONOS bridge formed between Cys95 and Lys131 of ISCU and Cys395 of Nsf1 (pdb code 6WI2, also observed in 6WIH). Left panel: structural overview. Right panel: close-up of the redox switch site. Competitive refinements (SONOS bridge only, 2 separate NOS bridges, mixture of SONOS and NOS) indicate a mixture of the SONOS bridge (refined occupancy 60%) and the NOS bridge between Lys135 and Cys95 (refined occupancy 40%) for this dataset.

NOS bridges are observed in an amazing diversity of structural motifs and chemical variations (Figs. 1 and 2). First, we analyzed the distance distribution in sequence between the NOS bridge lysine and cysteine residues –Cys^*a*^–(X)_*n* – 1_–Lys^*a* + *n*^–, where *a* and *a* + *n* denote the corresponding sequence positions, and *n* denotes the sequence distance (Supplementary Fig. 4). This analysis revealed a Gauss-like distribution, with the largest fraction of NOS bridges observed for short sequence distances (*n* < |10|) and the smallest distance being two positions in sequence (*n* + 2) in intrastrand/strand-like structures (Fig. 2a). Other motifs included intrahelical NOS bridges (mostly *n* + 4; Fig. 2b), interstrand bridges in, for example, barrel structures (Fig. 2c), bridges connecting helices and strands (Fig. 2d) or intraloop bridges (Fig. 2e). Most NOS bridges are formed intramolecularly within one protein chain; in a few examples, we detected intermolecular cross-links, either as single bridges between two different proteins (A–B-type) or as an NOS ‘double bridge’ in homodimeric assemblies (A_2_-type), where the corresponding lysine and cysteine residues of each chain form two reciprocal NOS bridges (Figs.1 and 2f). In one case, an NOS bridge was observed for a cysteine that is alternatively engaged in a disulfide bridge, suggesting a ‘mixed’ disulfide-NOS redox switch site (Fig. 2g). In terms of the oxidation state of the NOS cysteine, we exclusively found mono-oxidized species (sulfenic acid equivalent); there was not a single case where the cysteine sulfur was in a higher oxidation state (bonded to more than one oxygen atom). However, we discovered that some protein lysines may form NOS bridges with two cysteines at the same time, constituting a branching SONOS bridge in which the lysine nitrogen is in a dioxidized nitro state, as shown for, for example, galectin-1, a redox-sensitive protein with numerous important biological functions^14^ (Figs. 1 and 2h and Supplementary Table 3). To the best of our knowledge, the SONOS group would be the first native branching cross-link between amino acids in proteins. Amino acid-derived redox cofactors, which are formed through cross-links between two and, in rare cases, three amino acids (for example, methionine–tyrosine–tryptophan) do not possess a single branching center as the lysine nitrogen atom of the SONOS group^15,16^. Other examples for proteins with SONOS bridges include the main protease (Mpro) from SARS-CoV-2 (Fig. 3), an important drug target for fighting the current coronavirus disease 2019 (COVID-19) pandemic^17,18^, and the human NSF1–ISCU complex that is central to Fe–S cluster biogenesis in mitochondria^19^ (Extended Data Fig. 5). In both cases, structures with either an NOS or a SONOS bridge of the same redox switch were detected, suggesting a stepwise oxidation (Fig. 1). While the initial oxidation (NOS) involved structurally proximal lysine and cysteine residues, the second oxidation (SONOS) involved, in both cases, a ‘mobile’ cysteine that structurally fluctuates between the active sites and the redox site (Fig. 3 and Extended Data Fig. 5).

### Catalytic requirements for NOS/SONOS bridge formation

**Extended Data Fig. 6:**
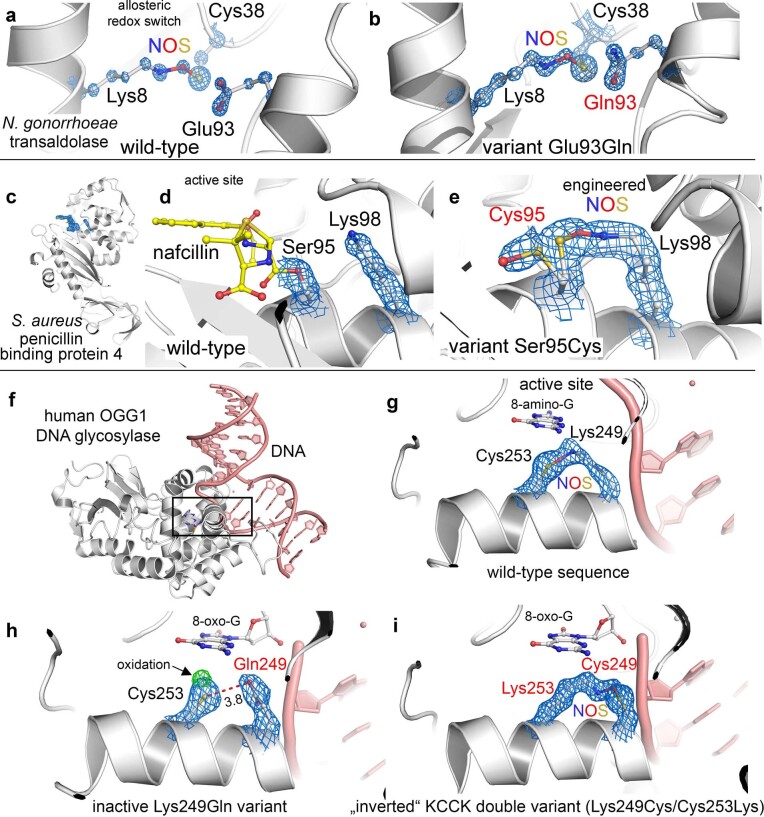
Structural requirements for formation and engineering of NOS bridges. Structural requirements for formation and engineering of NOS bridges. (**a**, **b**) Structure of the allosteric NOS bridge in *Neisseria gonorrhoeae* transaldolase wild-type (a, pdb code 6XZ4)^9^ and variant Glu93Gln (b, this study) showing NOS residues Lys8 and Cys38 as well as neighboring residue 93. The structural models of Lys8, Cys38 and Glu93 or Gln93 are superposed with the corresponding 2mFo-DFc electron density maps at 3σ. Note that NOS bridge formation does not require the presence of a catalytic residue at position 93. (**c**, **d**, **e**) An unintentionally engineered lysine-cysteine crosslink in *Staphylococcus aureus* penicillin binding protein 4 showing the overall protein structure (c) and a close-up of the active site with catalytic residues Ser95 (nucleophile) in linkage with nafcillin and Lys98 (d, pdb code 5TY2). In variant Ser95Cys (e), where the catalytic Ser95 has been replaced by cysteine, an NOS bridge was formed between introduced Cys95 and Lys98. The 2mFo-DFc electron density map is contoured at 1σ. In variant Ser95Cys, residue 95 was modeled in two alternative conformations. Note that a sulfenamide linkage with an N-S bond could also explain the density. (**f**-**i**) NOS bridges in the human DNA glycosylase OGG1. (**f**) Overall structure of OGG1 in complex with DNA (pdb code 1M3Q). (**g**) Close-up of the active site showing catalytic residue Lys249 (Schiff-base with DNA) and neighboring residue Cys253 forming an NOS bridge. The 2mFo-DFc electron density map is contoured at 1.5σ⊡ (**h**) Active site structure of inactive OGG1 variant Lys249Gln (pdb code 1EBM), in which the catalytic lysine had been replaced by glutamine. The 2mFo-DFc electron density map is shown in blue at 1.5σ and the mFo-DFc difference electron density map in green at 3σ⊡ Note that Gln249 and Cys253 form a H-bond rather than an NOS bridge and that Cys253 is likely to be oxidized as indicated by the positve peak in the difference electron density map. (**i**) Active site structure of OGG1 KCCK double variant Lys249Cys/Cys253Lys (pdb code 2XHI), in which the sequence positions of the NOS-bridge-forming Lys and Cys residues are inverted. The 2mFo-DFc electron density map is shown at 1.5σ. Note the presence of the NOS bridge in this ‘inverted’ variant akin to the wild-type sequence argueing against a mechanism, where catalysis is provided by neighboring residues for the NOS bridge to form.

In our initial discovery of the allosteric NOS redox switch in the enzyme transaldolase from *N. gonorrhoeae*^9^, we had speculated that a neighboring glutamate (Glu 93) might be catalytically essential for formation of the NOS bridge, akin to the suggested role of a glutamate in isopeptide bond formation in some proteins^20^ (Extended Data Fig. 6a). However, in many proteins with identified NOS or SONOS bridges, no amino acid capable of acid–base catalysis is found in the immediate vicinity of the redox switch, highlighting an intrinsic reactivity of structurally proximal lysine and cysteine residues, provided that ROS and/or oxygen is present. To test this hypothesis, we generated a transaldolase Glu93Gln variant in which a potential catalytic role of residue 93 is eliminated and analyzed the structure and enzymatic properties of the variant. The kinetic constants are very similar to the wild-type enzyme except for a slight 1.5-fold increase in the substrate Michaelis constant (Supplementary Table 4), and the variant remains redox activatable. Structural analysis of the Glu93Gln variant by X-ray crystallography (Supplementary Table 1) clearly indicates formation of the NOS bridge, arguing against a compulsory catalytic mode involving a neighboring amino acid (Extended Data Fig. 6b). The NOS cross-link is the only modification observable under non-reducing conditions, akin to our findings for the wild-type enzyme^9^, strongly suggesting that this switch controls the enzymatic activity. The mechanistic proposal of a non-protein-catalyzed NOS bridge formation is also supported by the finding that a lysine–cysteine cross-link was unintentionally engineered into penicillin-binding protein 4 from *Staphylococcus aureus*, where the catalytic nucleophile Ser 95 at the active site had been replaced by a cysteine that forms a cross-link with the cocatalytic Lys 98 (Extended Data Fig. 6c–e); however, a sulfenamide species cannot be excluded in that case as, for example, reported for a related engineered protein^21^. Likewise, a positional inversion of the NOS bridge-forming lysine and cysteine residues in the human DNA repair enzyme OGG1 resulted in the formation of an NOS bridge akin to the wild-type configuration (Extended Data Fig. 6f–i). While amino acid or enzyme catalysts are not compulsory for NOS bridge formation in vitro, they could impact the kinetics of the reaction. Also, we cannot exclude enzyme or metal ion catalysis under in vivo conditions, as in the case of disulfide bonds^22^.

### Chemical functions of NOS lysine and cysteine residues

**Fig. 4:**
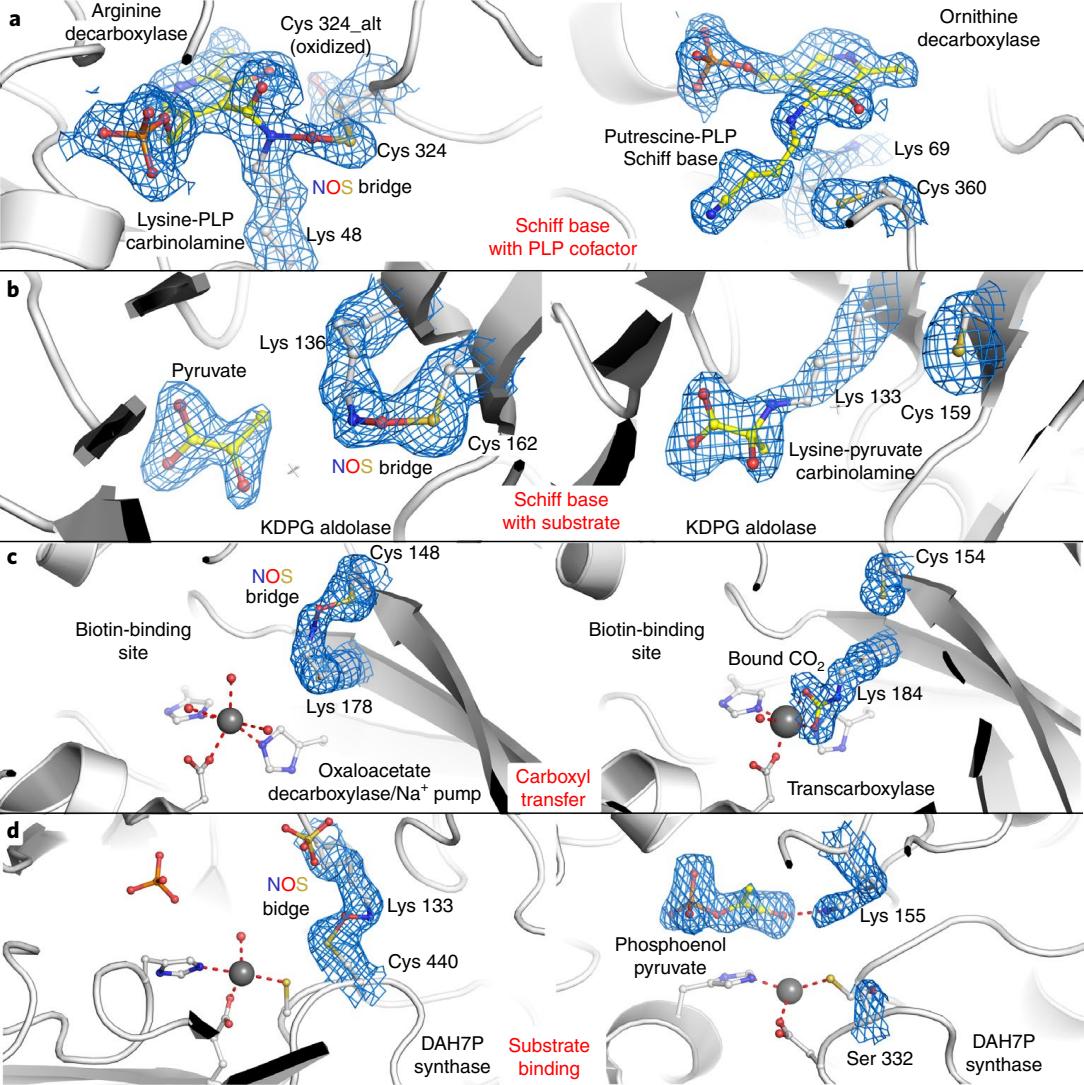
Functional roles of NOS bridge lysines in enzyme catalysis and in binding of enzymatic substrates or effectors. Left, proteins in the oxidized state with the NOS bridge present; right, same or closely related protein in the reduced state with the lysine exerting its function. The corresponding 2*mF*_o_–*DF*_c_ electron density maps are shown in blue at a contour level of 1*σ*. **a**, Catalytic lysines forming Schiff base intermediates in PLP-dependent enzymes; left, arginine decarboxylase from *Paramecium bursaria* chlorella virus (PDB: 2NV9), in which the catalytic Lys 48 forms an NOS bridge with Cys 324; right, ornithine decarboxylase from *T. brucei* in covalent complex with product putrescine (PDB: 1F3T). Note that in the presence of the NOS bridge, the reaction does not proceed beyond the carbinolamine. **b**, Catalytic lysines forming Schiff base intermediates with enzymatic substrates; left, KDPG aldolase from *Oleispira antarctica* in non-covalent complex with substrate pyruvate and with an NOS bridge between Lys 136 and Cys 162 (PDB: 3VCR); right, KDPG aldolase from *Escherichia coli* in covalent complex with substrate pyruvate (PDB: 1EUA). Note that in the presence of the NOS bridge, covalent catalysis is inhibited. **c**, Catalytic lysines in carboxyl transfer; left, oxaloacetate decarboxylase/Na^+^ pump from *Vibrio cholerae* with an NOS bridge between Lys 178 and Cys 148 (PDB: 2NX9); right, transcarboxylase 5S subunit from *Propionibacterium freudenreichii* with carboxylated Lys 184 (PDB: 1RQB) thought to be an intermediate in CO_2_ transfer to biotin. Note that in the presence of the NOS bridge, catalysis is inhibited. **d**, Lysines with roles in non-covalent binding of enzymatic substrates; left, DAH7P synthase from *Mycobacterium tuberculosis* (PDB: 3RZI) with an NOS bridge between Lys 133 and Cys 440; right, DAH7P synthase from *Listeria monocytogenes* in non-covalent complex with substrate phosphoenolpyruvate (PDB: 3TFC). This enzyme contains a serine (Ser 332) at the equivalent position of Cys 440 from *M. tuberculosis* DAH7P synthase and can therefore not form an NOS bridge. In the absence of the NOS bridge, the lysine forms a hydrogen bond with the carboxylate moiety of substrate phosphoenolpyruvate.

**Fig. 5:**
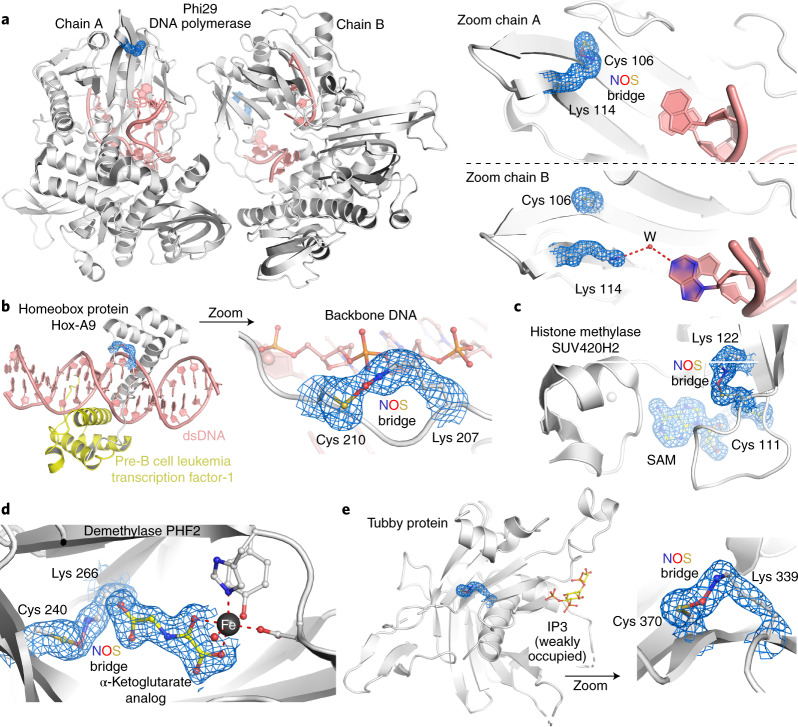
Proteins with NOS bridges inferred in DNA binding and transcription regulation. The corresponding 2*mF*_o_*–DF*_c_ electron density maps are shown in blue at a contour level of 1*σ*. **a**, DNA polymerase from *Bacillus* virus phi29 complexed with single-stranded DNA (PDB: 2PY5). Left, overall structure showing the two copies (chains A and B) in the asymmetric unit. The single-stranded DNA is highlighted in salmon red. Right, close-up of the NOS redox switch with residues Lys 114 and Cys 106. Note that in chain A, both residues form an NOS bridge (left), which is absent in chain B (right). In the absence of the NOS bridge, Lys 114 forms a hydrogen bond with a DNA base via a water molecule. **b**, Homeobox protein Hox-A9 from *Mus musculus* in complex with human pre-B cell leukemia transcription factor-1 and double-stranded DNA (dsDNA; PDB: 1PUF). Left, overall structure of the complex. The two proteins are colored individually, and the DNA is shown in salmon red. Right, close-up of the NOS bridge formed between Lys 207 and Cys 210 of Hox-A9. Note that the NOS bridge is at the binding interface with the backbone of the DNA, suggesting that Lys 207 in the reduced state interacts with the phosphate groups. **c**, Human histone-lysine *N*-methyltransferase SUV420H2 in complex with substrate *S*-adenosyl methionine (SAM) and with an NOS bridge formed between Lys 122 and Cys 111 (PDB: 3RQ4). Note the proximity of the NOS bridge with respect to the SAM-binding locale. **d**, Human demethylase PHF2 in complex with an analog of the α-ketoglutarate cofactor and with an NOS bridge formed between Lys 266 and Cys 240 (PDB: 3PU8). Note the proximity of the NOS bridge with respect to the cofactor-binding locale, suggesting a direct interaction of Lys 266 with the carboxylate moiety of the cofactor. **e**, Tubby protein from *Mus musculus* in complex with inositol trisphosphate (I3; PDB: 1I7E). Left, structural overview highlighting the ligand-binding site and the allosteric NOS switch. Right, close-up of the NOS bridge between Lys 339 and Cys 370.

**Extended Data Fig. 7:**
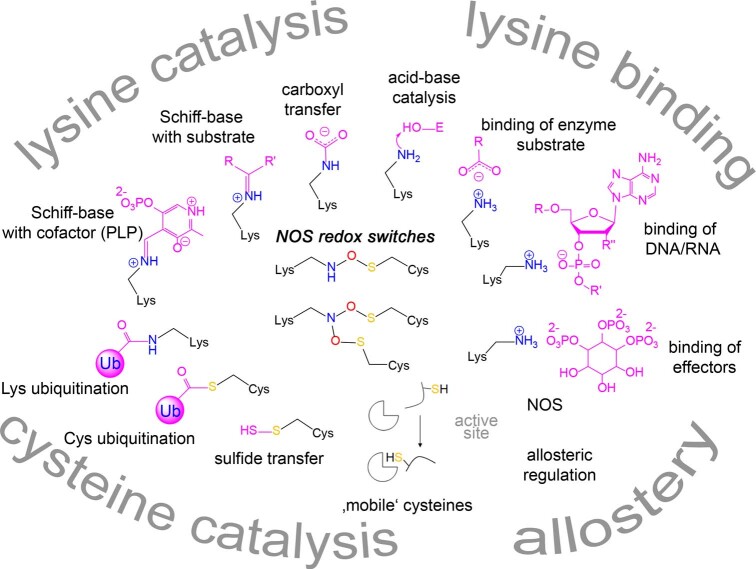
Chemical functions of lysine and cysteine residues forming NOS and SONOS bridges in proteins. Chemical functions of lysine and cysteine residues forming NOS and SONOS bridges in proteins. Four major functional categories could be identified including i) lysines with catalytic roles in enzyme mechanisms, ii) lysines involved in binding of enzyme substrates, nucleic acids and effectors, iii) cysteines with catalytic roles in enzyme mechanisms and iv) allosteric bridges, which are located remotely relative to the active/functional site. Structures of key reaction intermediates and interaction partners are highlighted. Note that these functions are exerted under reducing conditions that is in the absence of NOS/SONOS bridges. Formation of the NOS or SONOS bridge under oxidizing conditions leads to either a loss-of-function (catalytic lysines, catalytic cysteines), diminished biologial activity (lysine with binding roles) or modulated function (allosteric switches). Specific information about all proteins regarding origin, biological function, type of NOS/SONOS redox switch, suggested mechanism of the redox switch and potential relevance in disease states is compiled in Supplementary Tables 2 and 3.

**Extended Data Fig. 8:**
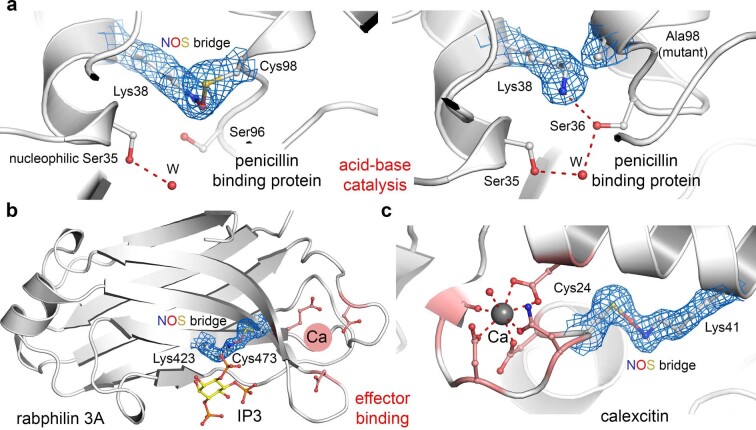
Functional roles of NOS bridge lysines in enzyme catalysis and in binding of enzymatic substrates or effectors (see also Fig. 4). Functional roles of NOS bridge lysines in enzyme catalysis and in binding of enzymatic substrates or effectors (see also Fig. 4). The corresponding 2mFo-DFc electron density maps are shown in blue at a contour level of 1σ. (**a**) Catalytic lysines acting as acid-base catalysts. Left: Penicillin binding protein (transpeptidase) from *Streptomyces sp*. K15 with an NOS bridge between Lys38 and Cys98 (pdb code 1SKF). Right: Penicillin binding protein variant Cys98Ala from *Streptomyces sp*. K15 (pdb code 1ES3). As Lys38 is thought to activate the nucleopilic Ser35 by acid-base catalysis, formation of an NOS bridge under oxidizing conditions is likely to inhibit catalysis. (**b,c**) Lysines with roles in binding of effectors. (**b**) Rabphilin 3a from *Rattus norvegicus* in complex with effector inositol trisphosphate (IP3) and with an NOS bridge formed between Lys423 and Cys473 (pdb code 4NP9). Ligand IP3 is weakly occupied with traceable density for the phosphate portions only. Lys423 is directly located at the binding site of IP3. The proximal Ca^2+^ binding site is indicated. (**c**) Calexcitin from *Loligo pealeii* with an NOS bridge formed between Lys41 and Cys24 proximal to the Ca^2+^ binding site (pdb code 2CCM).

**Extended Data Fig. 9:**
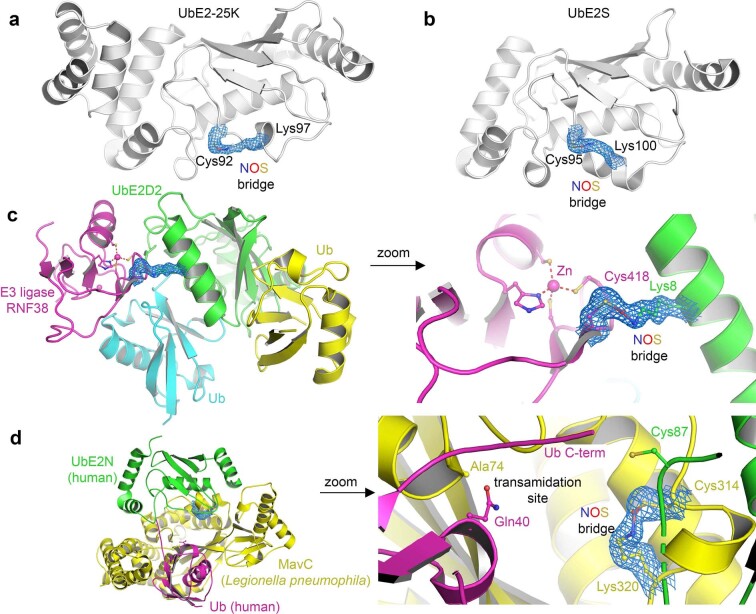
NOS bridges in proteins from the ubiquitin system. NOS bridges in proteins from the ubiquitin system. The corresponding 2mFo-DFc electron density maps are shown in blue at a contour level of 1σ. (**a**) Human ubiquitin-conjugating enzyme E2-25K (UbE2-25K) with an NOS bridge formed between catalytic residue Cys92 and Lys97, a potential auto-ubiquitination site (pdb code 3E46). (**b**) Human ubiquitin-conjugating enzyme E2-S (UbE2S) with an NOS bridge formed between catalytic residue Cys95 and Lys100, a potential auto-ubiquitination site (pdb code 6QHK). (**c**) Complex between ubiquitin-conjugating enzyme E2 D2 (UbE2D2), E3 ring ligase RNF38 and two ubiquitin (Ub) molecules (pdb code 4V3L). Left: Overall structure of the complex showing the proteins in individual colors. Right: Close-up of the intermolecular NOS bridge formed between Lys8 of E2 and Cys418 of E3. Note the proximity of the NOS bridge with respect to the Zn^2+^-binding site of E3. (**d**) Structure of a complex between MavC from *Legionella pneumophila* in complex with human ubiquitin-conjugating enzyme E2 N (UbE2N) and human ubiquitin (Ub) (pdb code 6ULH). MavC is a bacterial effector that inactivates the human ubiquitin system by catalyzing a transamidation reaction between Lys92 of UbE2N and Gln40 of Ub forming a dead-end complex^69^. Left: Overall structure of the complex showing the proteins in individual colors. Right: Close-up of the intramolecular NOS bridge in MavC formed between Lys320 and Cys314. Note the proximity of the NOS bridge with respect to the transamidation site.

Next, we analyzed the putative chemical functions of the lysine and cysteine residues forming the NOS/SONOS redox switches in the context of the respective protein’s structure and function. In the previously reported redox-sensitive transaldolase, the NOS bridge functions as an allosteric switch that changes the structure of the protein, including that of the active site, and thereby regulates enzymatic activity^9^. The operational modes of the NOS/SONOS redox switch residues in the now identified proteins are amazingly diverse and can be classified into several major classes, including lysines with direct catalytic roles, lysines with direct binding roles, cysteines with direct catalytic roles and allosteric switches (Fig. 4 and Extended Data Fig. 7). The manifold chemical tasks associated with the NOS lysine residues are particularly intriguing and include the full spectrum of lysine roles in protein function. NOS bridges were identified for proteins with catalytic lysines that form (1) Schiff bases with the pyridoxal phosphate (PLP) cofactor in PLP-dependent enzymes (for example, arginine/ornithine decarboxylase), (2) Schiff bases with enzymatic substrates (for example, KDPG aldolase), (3) carbamate intermediates in covalent CO_2_ transfer to biotin (for example, oxaloacetate decarboxylase/Na^+^ pump) and lysines that (4) act as acid–base catalysts in the active site of enzymes (for example, penicillin-binding protein; Fig. 4a–c and Extended Data Fig. 8a). Apart from these catalytic roles, numerous proteins were identified where the NOS lysine is directly involved in binding of enzymatic substrates (for example, DAH7P synthase) or of effectors, such as inositolphosphates (for example, rabphilin), often interacting with a negatively charged moiety of substrate or effector (Fig. 4d and Extended Data Fig. 8b). In other cases, the NOS bridge is located proximal to regulatory metal-binding sites (for example, Ca^2+^; Extended Data Fig. 8c). An interesting observation concerns the involvement of the NOS bridge lysines in direct binding of DNA. In, for example, DNA polymerase, the lysine interacts with the base moiety of single-stranded DNA (Fig. 5a), while in homeobox protein Hox, it binds to the phosphate backbone of double-stranded DNA (Fig. 5b). Interestingly, NOS redox switches are also found for histone writers and erasers as well as for the transcription factor tubby, suggesting a critical and multilayered role of these switches for the regulation of gene expression (Fig. 5c–e). Catalytic cysteines were detected for several ubiquitin E2 ligases (Extended Data Fig. 9) and the Fe–S cluster biogenesis complex ISCU–NSF1 (Extended Data Fig. 5), as discussed before.

### Possible biological functions of NOS and SONOS switches

The putative NOS and SONOS bridges detected in the crystallographic protein structures were apparently formed due to the presence of oxygen. The responsible oxidants in vivo need be identified, but it seems reasonable to suggest that NOS/SONOS bridges form under oxidizing conditions as, for example, oxidative stress by ROS^1–6^. However, these switches might also function as sensors for oxygen. Some of the species with NOS bridge-containing proteins are facultative anaerobic organisms (*V. cholerae* and *N. gonorrhoeae*), and the adaption to anaerobic or aerobic conditions entails reprogramming on different cellular levels of metabolism, signaling, gene expression and so on. Also, interestingly, our analysis identified NOS bridges in histone demethylases, which were recently reported to be oxygen dependent in a HIF-independent manner^23^.

Our previous studies had indicated that the NOS bridge is a reversible redox switch that prevents overoxidation of the involved cysteine residues, and a similar function can be envisaged for the here newly identified switches. Formation of NOS/SONOS bridges in proteins, where the lysine and cysteine residues have direct roles in catalysis, will go along with a reversible loss of function. In case of direct roles in the binding of enzymatic substrates, effectors or DNA, NOS bridge formation would lead to a reduced affinity and with that a diminished biological activity, although the extent may vary depending on the structural context. For allosteric redox switches, both loss-of-function as well as gain-of-function scenarios seem possible with a variable modulation of biological function. In support of this suggested function, the available biochemical data for several key redox-sensitive proteins with identified NOS/SONOS redox switches bearing catalytic lysines or cysteines indeed indicate a reversible loss of function in vivo under oxidative stress conditions (reaction with oxidants hydrogen peroxide or diamide) that is related to cysteine oxidation, as in the case of, for example, DNA repair enzyme OGG1 (Schiff base lysine)^24^, ornithine decarboxylase (catalytic lysine and cysteine)^25^ or ubiquitin E2 ligases (catalytic cysteine)^26^. In the case of ornithine decarboxylase, a mutation of the NOS cysteine made the resultant protein variant redox insensitive, supporting a regulatory in vivo function. For numerous other cases, it has been established under in vitro conditions that either reducing conditions are required for full biological activity or that oxidizing conditions deteriorate protein activity^9,27–29^. In the case of galectin-1, which possesses an allosteric SONOS switch, it was established that the protein exerts different biological functions under oxidizing and reducing conditions in vivo and that binding of effectors requires the reduced state^30,31^. For the human protein, exactly these two cysteines engaged in forming the SONOS bridge in the *R. norvegicus* ortholog (Fig. 2h) were identified as the redox switch site and suggested to form a disulfide, although the latter could not be directly demonstrated^31^. The potential role of NOS redox switches in regulating DNA binding might provide a mechanism for the well-established redox-dependent action of many transcription factors^8,32^. A putative regulatory role of the detected NOS/SONOS redox switches is further supported by the high degree of conservation of the lysine–cysteine pairs in the protein orthologs (either across different domains of life or in clades of related organisms) despite the fact that in many proteins, only one of the two residues has a direct functional role required for biological activity, as reported for, for example, OGG1 or rabphilin (Supplementary Figs. 5 and 6)^33,34^.

### NOS/SONOS bridges in proteins from pathogens and humans

We further classified the proteins with NOS/SONOS switches regarding their biological function. First, we analyzed proteins from human and plant pathogens, including viruses, bacteria and parasites (Supplementary Fig. 7 and Supplementary Table 2). In the case of pathogenic human viruses, NOS switch-containing proteins with essential roles for all critical phases of the virus life cycle, such as infection (adenovirus), replication (SARS-CoV-2) and maturation assembly (cytomegalovirus), were detected. In bacteria and parasites, we identified numerous essential biosynthetic enzymes (amino acid, isoprenoid and polyamine biosynthesis), Na^+^ transporters required for ATP biosynthesis, transcription regulators, virulence factors and proteins in the context of pathogen–host interactions. Remarkably, numerous proteins originate from some of the world’s most dangerous pathogens, including, among others, SARS-CoV-2 (COVID-19), *M. tuberculosis* (tuberculosis), *V. cholerae* (cholera), *Pseudomonas aerigunosa* (pneumonia), *S. aureus* (bacterial superinfections), *Legionella pneumophila* (Legionnaires’ disease) or *Trypanosoma* species (Chagas disease, African sleeping sickness). NOS bridges were also discovered in major plant pathogens, including *X. campestris* and *X. axonopodis*, the causative agents of ‘black rot’ in cruciferous vegetables and bacterial pustule of soybean^35^.

In humans, NOS redox switches are found in different protein families with established roles in the oxidative stress response, redox signaling and homeostasis (Supplementary Fig. 7 and Supplementary Table 3). Interestingly, many proteins are localized in the nucleus or membrane (Supplementary Data 1). Major families comprise proteins in DNA repair (excision of oxidized bases, cleavage of protein–DNA cross-links), in transcription regulation (sensing and binding of DNA, histone writers and erasers), in ribosomal protein translation (translation factors), in protein degradation (ubiquitin E2 ligases, E3 ring ligase), in signaling (various kinases, neurotransmission, inositolphosphate signaling) and in the biosynthesis of redox-sensitive cofactors and rare amino acids (Fe–S cluster, SAM and selenocysteine; see examples in Figs. 2 and 5 and Extended Data Figs. 5 and 9). Also, the enzyme selenophosphate synthetase 1 was identified (Fig. 2e), which is known to be an essential factor of cellular redox homeostasis by regulating the expression levels of, for example, glutaredoxin and glutathione transferase, which protect cells from oxidative stress^36^. Many of the identified human proteins have been inferred in severe diseases, including various cancers, Alzheimer’s disease, Parkinson’s disease, obesity, autoimmune diseases and others (Supplementary Table 3). The discovery of the naturally evolved NOS redox switches may therefore unlock new therapeutic directions in plenty of disease states by addressing or manipulating the switch site. Prominent examples are the Mpro from SARS-CoV-2 (refs. ^17,18^) or human focal adhesion kinase, which are validated drug targets for treatment of COVID-19 and invasive cancers^37^, respectively.

## Discussion

NOS and SONOS redox switches are ubiquitous regulatory elements in proteins that reversibly alter protein function in response to changing redox conditions as, for example, under oxidative stress. The involvement of lysines with direct roles in enzyme catalysis and/or in binding of enzymatic substrates, nucleic acids and effectors expands the chemical repertoire of organisms to deal with changing redox conditions and constitutes a new general regulatory principle in biology. Apart from new directions in medical applications, the identified design principles of naturally evolved NOS/SONOS switches are likely to inspire peptide and protein design^38^; in particular, the newly found branching SONOS cross-link bears great potential. The potential existence of sulfenamide species as another form of lysine–cysteine redox switches needs to be further explored^21,39^.

## Methods

### Experimental procedures

#### General information

The protein concentration of *N. gonorrhoeae* transaldolase (*Ng*TAL) was determined by UV/Vis spectroscopy by measuring the absorption at a wavelength of 280 nm and using the molar extinction coefficient (*ε*_*Ng*TAL_ = 28,420 M^–1^ cm^–1^) determined according to Gill and Hippel^40^.

#### Mutagenesis, protein expression and purification

For expression of *Ng*TAL (UniProtID: Q5F6E9), we used a pET SUMO vector, as recently described^9^. Mutant strains were generated by site-directed mutagenesis PCR using the QuikChange site-directed mutagenesis protocol (Stratagene). We used the following primer pair: *Ng*TAL variant Glu93Gln forward, 5′-GTCTGGCACAACATGAAAGCAC-3′, and reverse, 5′-GTGCTTTCATGTTGTGCCAGAC-3′. Expression and purification were performed as recently reported^9^.

#### Steady-state kinetic analysis

Steady-state kinetic analysis of *Ng*TAL wild type was performed using a coupled enzymatic assay that monitors the conversion of ketose donor d-fructose-6-phosphate and aldose acceptor d-erythrose-4-phosphate into products sedoheptulose-7-phosphate and glyceraldehyde-3-phosphate at a wavelength of 340 nm (refs. ^41,42^). Measurements were conducted for both reducing and non-reducing conditions. For measurements under reducing conditions, the protein stock was supplemented with 20 mM dithiothreitol (DTT), resulting in a final concentration of 1 mM DTT in the assay mix. Initial rates were estimated by either linear regression of the absorbance signal over the first 5 s of the measurements or, if substrate activation was observed, by using equation (1):

1 $$A_{340}(t) = A_0 - {\Delta}ss \cdot t + \frac{{{\Delta}ss - {\Delta}_0}}{{k_{{{{\mathrm{obs}}}}}}} \cdot \left[ {1 - exp^{( - k_{{{{\mathrm{obs}}}}} \cdot t)}} \right]$$

Here, *A*_0_ denotes the starting absorbance at 340 nm, Δ*ss* denotes the absorbance change at established steady state (steady-state rate), Δ_0_ denotes the absorbance change at *t* = 0 (initial rate) and *k*_obs_ denotes the first-order rate constant of activation for the transition into the fully active enzyme form.

Thus, obtained steady-state activities were analyzed using the Michaelis–Menten equation and Hill equation.

#### Crystallization, X-ray data collection, processing and model building

*Ng*TAL wild type and variant Glu93Gln were crystallized and cryoprotected, as detailed in ref. ^9^. Diffraction data of single protein crystals were collected using synchrotron radiation at beamline P14 of DESY EMBL, Hamburg, Germany, at a wavelength of either 0.9763 Å (*Ng*TAL wild type, dose series) or 0.689 Å (*Ng*TAL variant Glu93Gln) at 100 K using an EIGER 16M detector. For crystals of *Ng*TAL wild type, we conducted a dose-series experiment, depositing-defined doses (0.27–5.4 MGy in 0.27-MGy increments over 360° using identical start positions; the diffraction-weighted doses were calculated using RADDOSE-3D^43^) per dataset. For data processing, the XDS package was used^44^. Subsequent refinement and model building was performed using PHENIX.REFINE^45^ and COOT^46^. Phasing was performed by rigid body refinement using our previously determined *Ng*TAL structure (PDB: 6ZX4) as an initial model. For initial coordinate disturbance, truncated datasets were subjected to simulated annealing in PHENIX.REFINE using a value of 7,000 K as start_temperature. The resulting maps were blurred to simulate more realistic B-factors for the selected resolutions^47^ (1.0: 15; 1.5: 25; 2.0: 30; 2.5: 40; 3.0: 65). The geometry of the structure was validated using MolProbity^48^. Representations of structures were prepared using PyMOL (Schrödinger, The PyMOL Molecular Graphics System, version 1.8.). The Ramachandran statistics are 98.85% in the favored and 1.15% in the allowed region for 7ODO, 98.85% in the favored and 1.15% in the allowed region for 7ODP, 98.85% in the favored and 1.15% in the allowed region for 7ODQ and 98.55% in the favored and 1.45% in the allowed region for 7OEY.

#### Computational methods

We performed a series of electronic structure calculations to sample the conformational space and the relative energy of a model system consisting of a single lysine and cysteine residues. The residues were truncated at the α-carbon, with the latter represented as a methyl group to reduce the influence of electrostatics in the terminal moieties, closer to what is to be expected from a protein backbone. Three different bonding situations were sampled. The first corresponds to a neutral lysine interacting with a cysteine (NHS). The second system considered was a protonated lysine with a cysteine (NHS^+^). Both cases will build close contacts through hydrogen bonds, either with the lysine as donor (NHS^+^) or the cysteine (NHS). In the last system, we considered the covalently bound residues, with the lysine nitrogen and the cysteine sulfur bonding with an intercalated oxygen atom (NOS).

In a first set of calculations, we studied the dependence of the N–S distances on the dielectric constant. To this purpose, a representative conformer for each of the systems was optimized in different solvents (vacuum: *ε* = 0.0; diethylether: *ε* = 4.2; acetone: *ε* = 20.5; methanol: *ε* = 32.6; 1,2-ethanediol: *ε* = 40.2; dimethylsulfoxide: *ε* = 46.8; water: *ε* = 78.4) using the SMD^49^ solvation model. The software package Gaussian16-A.03 (Gaussian 16, revision C.01) was used for the aforementioned calculations, with the B3LYP-D3(BJ)/def2-SVP^50–53^ level of theory. A constrained α-carbon distance of 10 Å was applied for all the calculations with different solvents. The results are provided in Supplementary Fig. 1. The only difference observed was a smaller difference between the distances observed in the NHS and NHS^+^ systems. Considering the stable behavior of the N–S distances in different environments, the following calculations were performed in vacuum for simplicity and for a more straightforward generalization.

Three different α-carbon distances (6, 8 and 10 Å) between lysine and cysteine were applied for the extended sampling runs (the results are provided in Extended Data Fig. 1). Initial conformer sampling was performed using CREST-2.10.2 (ref. ^54^), whereby the conformer optimization was conducted with xTB-6.4.0 (ref. ^55^) using the semiempirical tight binding-based quantum chemistry method GFN2-xTB^56^ with the ‘extreme’ optimization criteria (*E*_conv_ = 5 × 10^−8^ Hartree) and the ‘NCI’ option.

After initial global sampling at the semiempirical level, the structures were refined in two stages at the density functional theory level. For NOS, the collected GFN2-xTB conformer structures were further optimized using B3LYP-D3(BJ)/def2-SVP^50–53^, again with the use of the Gaussian16-A.03 program package. Conformers with imaginary frequencies were directly abandoned and not used further for optimization or data statistics. For neutral and protonated hydrogen-bonded systems (NHS and NHS^+^), the conformers were additionally filtered to guarantee the existence of hydrogen bonds and, thereby, afford the closest contacts possible. Criteria of a distance shorter than 2.5 Å between the hydrogen (H) and the hydrogen acceptor (A) and an angle ADH (D: hydrogen donor) smaller than 30° were taken. Determination of the hydrogen atom for the building of the hydrogen bond follows the shortest distance among the potential hydrogen bonds between the amine and thiol residues.

The conformations obtained from B3LYP-D3(BJ)/def2-SVP were further sorted according to their electronic energies from low to high. If the energy difference between two conformations was larger than 0.1 kcal mol^–1^ and the root mean square difference (r.m.s.d.) for the superimposed structures was larger than 0.125 Å (ref. ^57^), the two conformations were considered to be unique and subject to further calculations. From this pool of structures, we considered the lowest-lying conformers (up to 2 kcal mol^–1^ in difference to the global minimum) and performed further optimizations at the B3LYP-D3(BJ)/def2-TZVPP level of theory. For both NHS and NHS^+^ systems, the additional filter process to verify the existence of hydrogen bonds was again applied. The results of the first stage (B3LYP-D3(BJ)/def2-SVP) and the second stage (B3LYP-D3(BJ)/def2-TZVPP) of optimizations were used and displayed in Extended Data Fig. 1. An overview of different geometric values and their correlation for the NOS bond are provided in Supplementary Fig. 2.

In a further set of calculations, we investigated what could be the lowest energy conformer for both NHS and NHS^+^ bearing an N–S distance of 2.7 Å. By comparing the results of this sampling, we were able to assess the energy penalty associated with bringing the two residues in such a close vicinity without any covalent bond formation. This involved two further sampling rounds with the α-carbon distances set to 8 Å. The latter choice was considered as non-critical, given that the relative energetic and geometric information for the different bonding situations did not change dramatically following variation of the said parameter. Each sampling run was performed following the same steps as before but with an added constraint to the N–S distance of 2.7 Å. No additional filter process for hydrogen bonds was applied. The r.m.s.d. criteria were also not applied because we were not interested in a distribution but simply the most energetically stable geometry. The energies of the lowest conformers (‘global’ minima) were compared for the runs with and without the distance constraint. This resulted in an energy gap of 4.3 kcal mol^–1^ (Supplementary Fig. 8). The thermochemical calculations for formation of the SONOS bridge are shown for the SARS-CoV-2 Mpro (Supplementary Fig. 9), which showcase a putative pathway for two subsequent oxidation steps.

#### Search of the PDB for potential NOS or SONOS bridges

On 2 December 2020, we searched all deposited structures at the PDB (www.rcsb.org) with a resolution of 2 Å or better (65,327 of ~170,000 structures) for potential NOS or SONOS bridges by using the program NCONT of the CCP4 suite^58^. In view of the quantum chemically calculated N–S interatomic distance of 2.6–2.7 Å for NOS bridges, we defined thresholds of 3.00 Å as an upper limit (exclusion of non-covalent interactions) and 2.45 Å as a lower limit (exclusion of sulfenamides with N–S bonds) for the search. As non-covalent hydrogen-bond interactions between lysines and cysteines exhibit interatomic distances of >3.2 Å, we considered a cutoff at 3 Å to be a robust discriminator between covalent and non-covalent cysteine–lysine interactions. A total of 285 PDB entries with ~400 potential NOS/SONOS bridges were identified; for 266 of these, the corresponding structure factors were deposited. We examined all entries manually and inspected the potential NOS/SONOS redox switch sites. We also calculated *mF*_o_–*DF*_c_ electron density OMIT maps to eliminate model bias using PHENIX.POLDER^59^, omitting all entities constituting the suggested bridge. In case the electron density maps indicated the presence of a covalent cross-link between the lysine N atom and the cysteine S atom, we rebuilt the structural model with an NOS/SONOS bridge and compared the obtained models and electron density maps with those calculated for structures with a non-covalent interaction between the lysine and cysteine side chains. Model building was performed using COOT^46^, and refinements were done with PHENIX.REFINE^45^.

#### Analysis of sequence conservation

The analysis of sequence conservation of lysine and cysteine residues forming NOS or SONOS bridges was performed for the 1,000 closest related protein sequences obtained using BLASTp^60^ for the respective protein of interest. Alignment was performed using MAFFT^61^.

#### Analysis of phylogenetic distribution of NOS bridge-containing proteins

Sequences of proteins identified to likely or possibly contain NOS/SONOS bridges (Supplementary Data 1) were downloaded from the PDB database. For each protein, homologs were searched with BLASTp^62^ against NCBI’s non-redundant database, using a stringent E value cutoff (<10^–25^). A custom Python script powered by ETE3 (ref. ^63^) was used to identify the taxonomic lineage of each hit by querying NCBI’s taxonomy database with each hit’s taxid. Results were summarized at the third hierarchical level of NCBI’s taxonomy and plotted, along with the taxonomic affiliation of proteins identified in PDB, onto the Tree of Life, according to recent studies^64–68^.

### Reporting Summary

Further information on research design is available in the Nature Research Reporting Summary linked to this article.

## Online content

Any methods, additional references, Nature Research reporting summaries, source data, extended data, supplementary information, acknowledgements, peer review information; details of author contributions and competing interests; and statements of data and code availability are available at 10.1038/s41589-021-00966-5.

## Supplementary information

Supplementary Information Supplementary Figs. 1–9 and Tables 1–4.

Reporting Summary

Supplementary Data 1 Excel-based data table listing all PDB entries (resolution of ≤2.0 Å) with close contacts between cysteine and lysine side chains (N_Lys_–S_Cys_ interatomic distance of <3 Å). The table contains the corresponding PDB entry, resolution, sequence positions of the lysine and cysteine residues, protein name and source (organism), cellular localization and identification and classification of NOS and/or SONOS cross-links.

## Data Availability

The refined structural protein models and corresponding structure–factor amplitudes are deposited under PDB accession codes 7OEY (*Ng*TAL variant Glu93Gln oxidized), 7ODO (*Ng*TAL wild type oxidized, 0.27-MGy dose), 7ODP (*Ng*TAL wild type oxidized, 2.7-MGy dose) and 7ODQ (*Ng*TAL wild type oxidized, 5.4-MGy dose). All structures cited in this publication are available under their respective PDB accession codes. All other data are available on request.
