# Supplementary material for: Widespread occurrence of covalent lysine–cysteine redox switches in proteins
Source: Nat Chem Biol. 2022 Feb 14;18(4):368–75. doi: 10.1038/s41589-021-00966-5 (PMC8964421; doi:10.1038/s41589-021-00966-5)
Supplement: Supplementary file 1 — Supplementary Figs. 1–9 and Tables 1–4. [file 41589_2021_966_MOESM1_ESM.pdf]

nature portfolio

https://doi.org/10.1038/s41589-021-00966-5

Supplementary information

Widespread occurrence of covalent lysine–cysteine redox switches in proteins

In the format provided by the
authors and unedited

Supplementary Information

Widespread occurrence of covalent lysine–cysteine redox switches in proteins

Fabian Rabe von Pappenheim [1,2] , Marie Wensien [1,2] , Jin Ye [3], Jon Uranga [3], Iker Irisarri [4,5], Jan de Vries [4,5], Lisa-Marie Funk [1,2], Ricardo A. Mata [3], & Kai Tittmann [1,2 *]

[1] Department of Molecular Enzymology, Göttingen Center of Molecular Biosciences, Georg-August University Göttingen, Julia-Lermontowa-Weg 3, D-37077 Göttingen, Germany

[2] Department of Structural Dynamics, Max-Planck-Institute for Biophysical Chemistry, Am Fassberg 11, D-37077 Göttingen, Germany

[3] Institute of Physical Chemistry, Georg-August University Göttingen, Tammannstraße 6, D-37077 Göttingen, Germany

[4] Institute for Microbiology and Genetics & Göttingen Center of Molecular Biosciences, Georg-August University Göttingen, Goldschmidtstrasse 1, D-37077 Göttingen, Germany;

[5] Campus Institute Data Science, Georg-August University Göttingen

[*] To whom correspondence shall be addressed: ktittma@gwdg.de (K.T.)

Supplementary Data

Supplementary Data 1 (caption)

Excel-based data table listing all pdb entries (resolution ≤ 2.0 Å) with close contacts between cysteine and lysine sidechains (N_{Lys} - S_{Cys} interatomic distance < 3 Å). The table contains the corresponding pdb entry, resolution, sequence positions of the lysine and cysteine residues, protein name and source (organism), cellular localisation as well as identification and classification of NOS and/or SONOS crosslinks.

Supplementary Figures

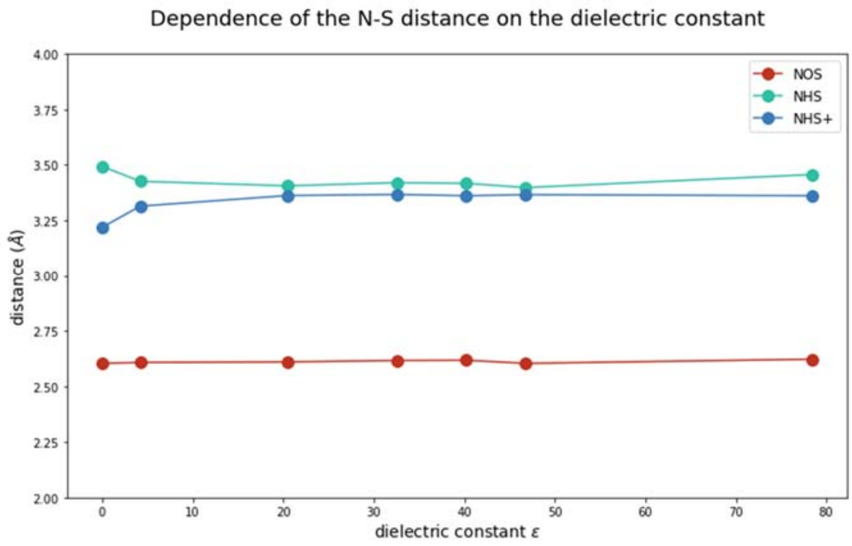

Supplementary Fig. 1. Dependence of the N-S distance on the dielectric constant for the three model situations of Lys-Cys binding. The calculations were carried out for a single sampled conformer at the B3LYP-D3(BJ)/def2-SVP level of theory applying different dielectric constants with the use of the SMD module.

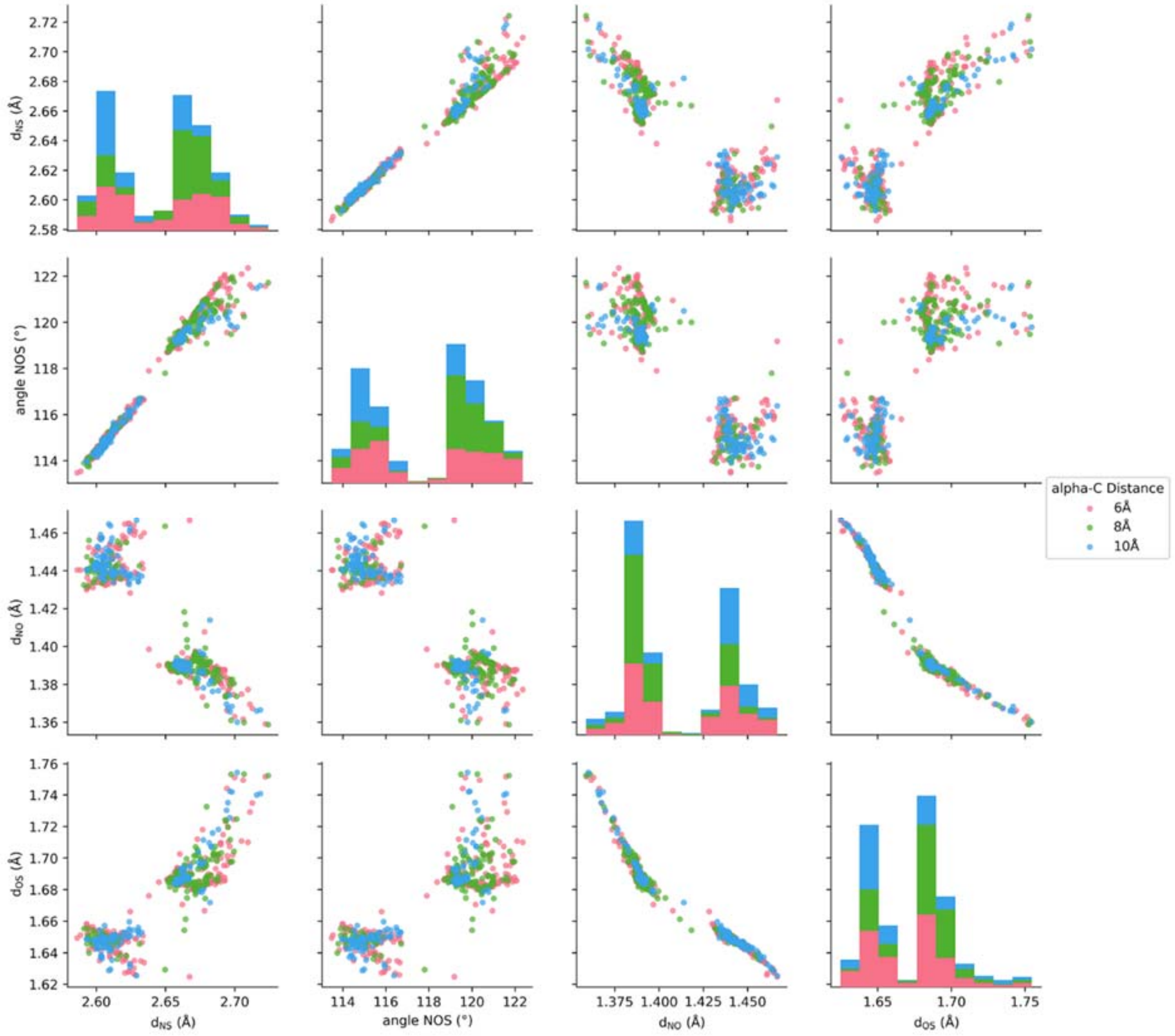

Supplementary Fig. 2. Geometric properties of NOS bridges. Pair plots for four selected structural variables of the NOS bond: N-S distance (dNS), NOS angle (Angle N-O-S), N-O distance (dNO) and O-S distance (dOS). The data used corresponds to the larger set of structures obtained with the def2-SVP basis set (the same as panels a and b). Several variables correlate strongly. The bimodal distribution of the N-S distances is due to steric constraints between the sulfur atom and the lysine chain, with an interplay between the N-O and the O-S bond distances. The N-O-S angle, as it should be expected, strongly correlates with the N-S distance. The results are largely independent of the distance between the α-carbons of the two chains.

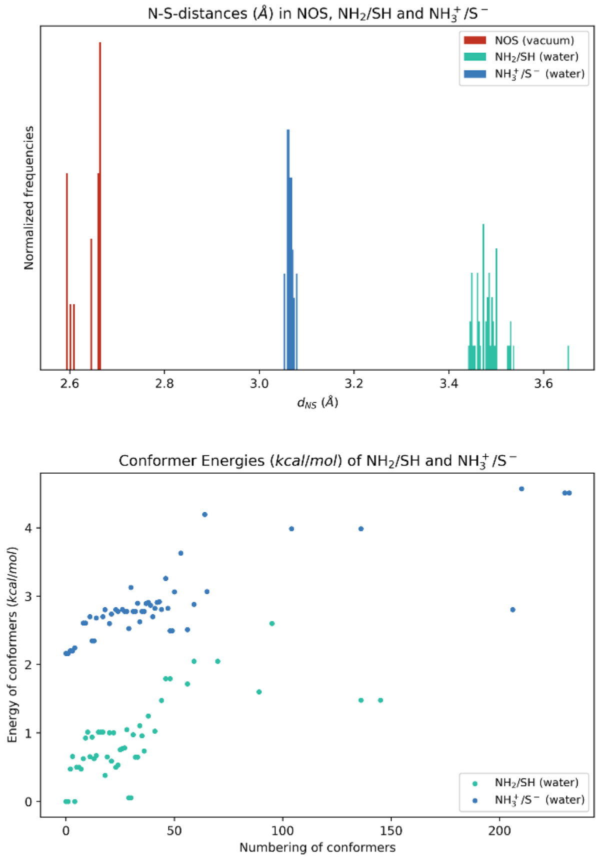

Supplementary Fig. 3. Top: histogram of N-S distances (normalised for each set individually), from model calculations on isolated Lysine and Cysteine residues (alpha-carbon distance of 8 Å). The data includes the NOS data (just as listed in Supplementary Fig. 1) adding the two tautomers for Lys(NH_2)/Cys(SH) and Lys(NH_3^+)/Cys(S^-), the latter data obtained with the use of the SMD continuum model with water as solvent. The geometries were optimised at the B3LYP-D3(BJ)/def2-TZVPP level of theory. Bottom: relative energy differences for the two non-covalent bonded sets, showing that the most stable structures are in the combination Lys(NH_2)/Cys(SH) (about 2 kcal/mol lower), even with such a high dielectric constant. The Lys(NH_3^+)/Cys(S^-) minima are not obtained for the models in vacuum.

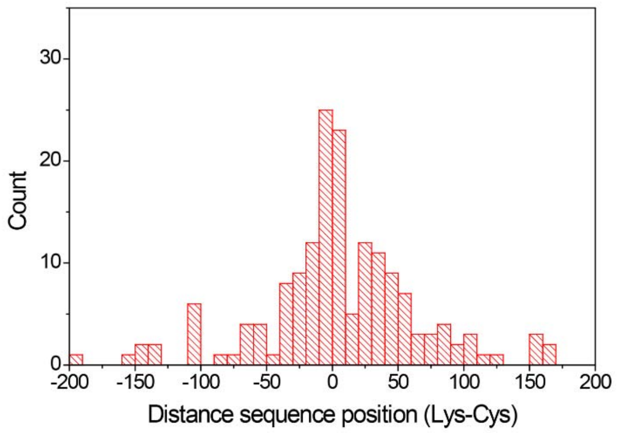

Supplementary Fig. 4. Histogram showing the distribution of detected proteins (non-redundant counts) containing lysine-cysteine crosslinks in dependence from the distance in sequence between the lysine and cysteine residues.

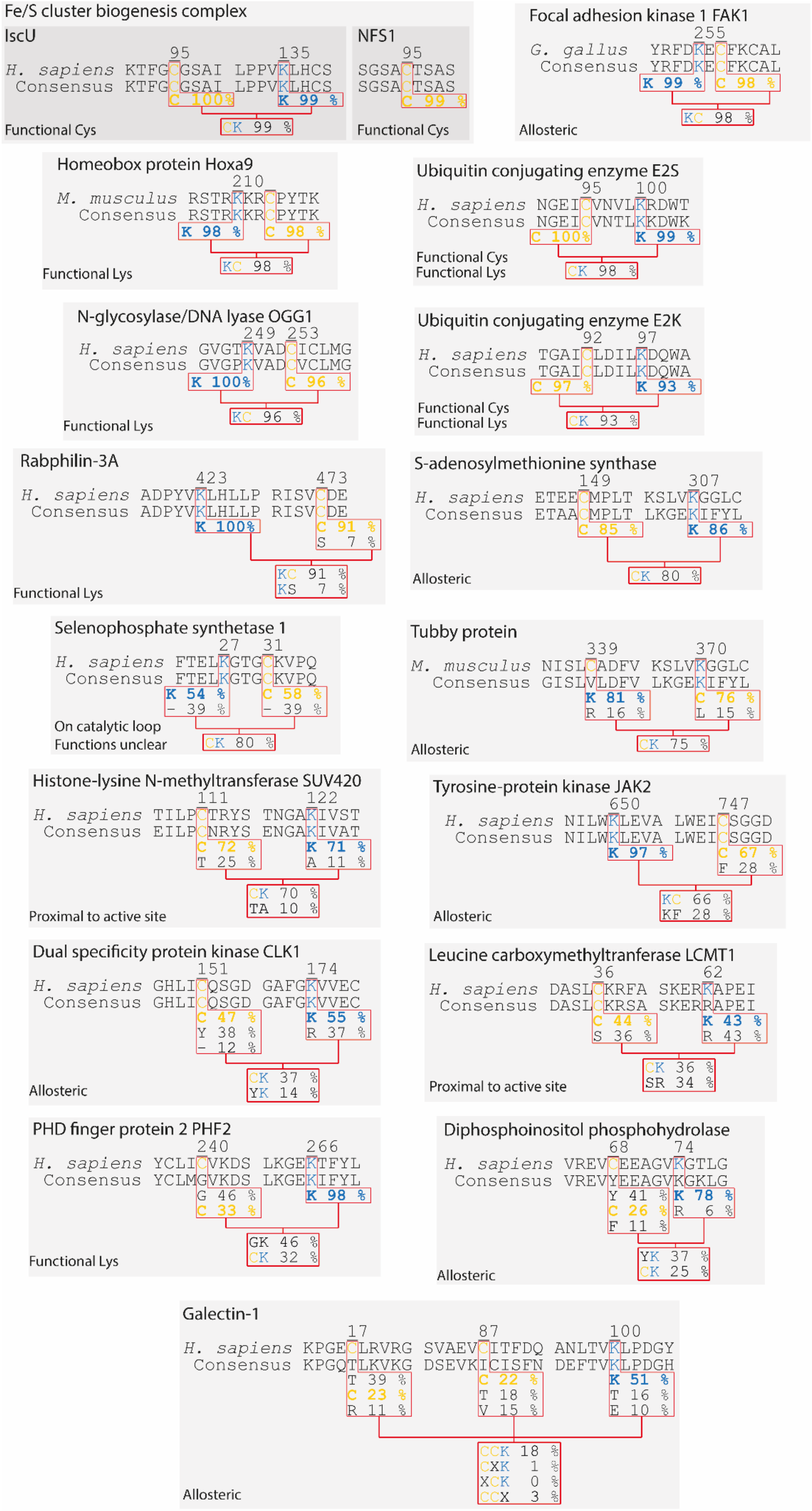

Supplementary Fig. 5. Sequence conservation of NOS bridge residues identified in human proteins or from model organisms.

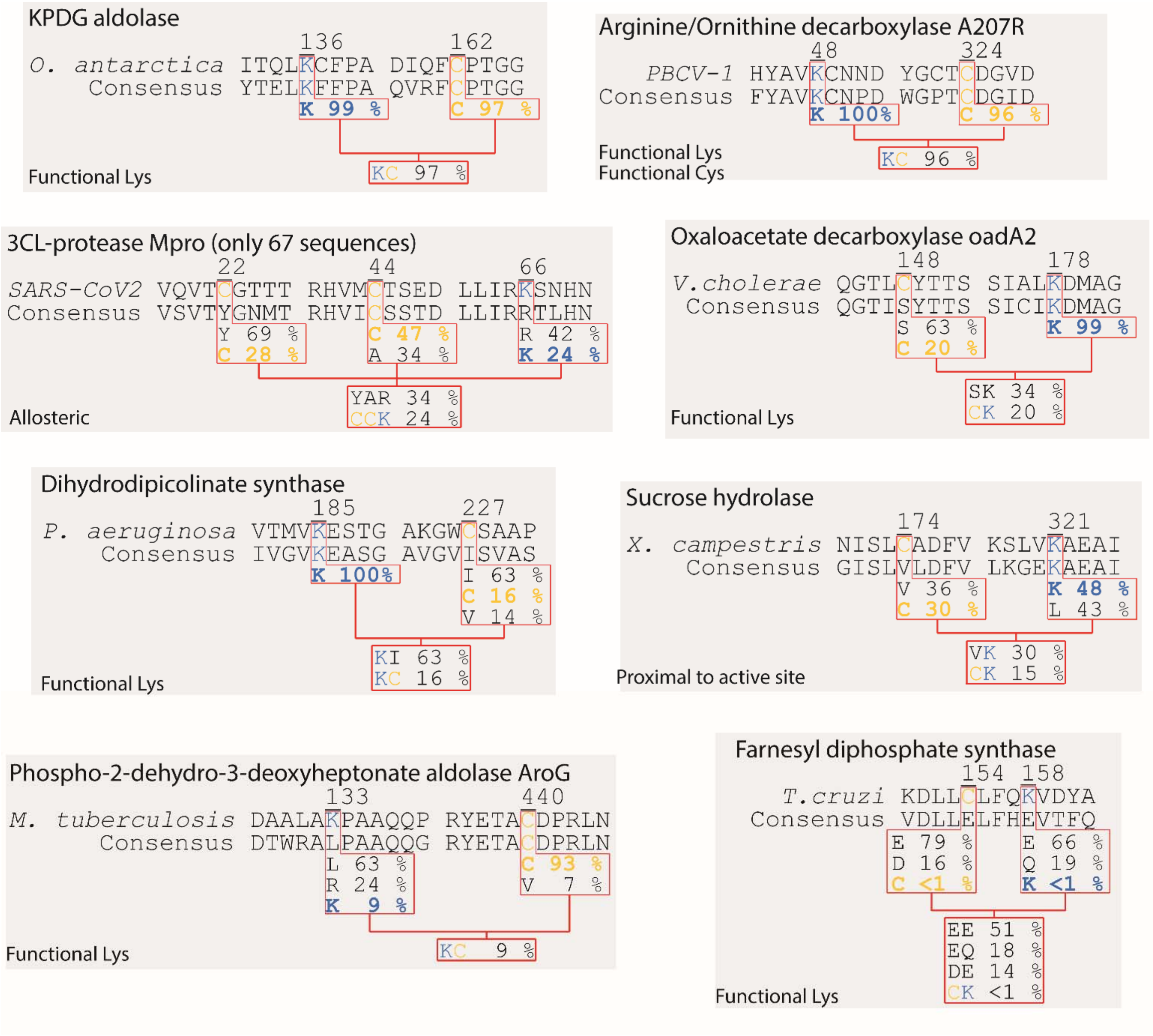

Supplementary Fig. 6. Sequence conservation of NOS bridge residues identified in proteins from pathogens.

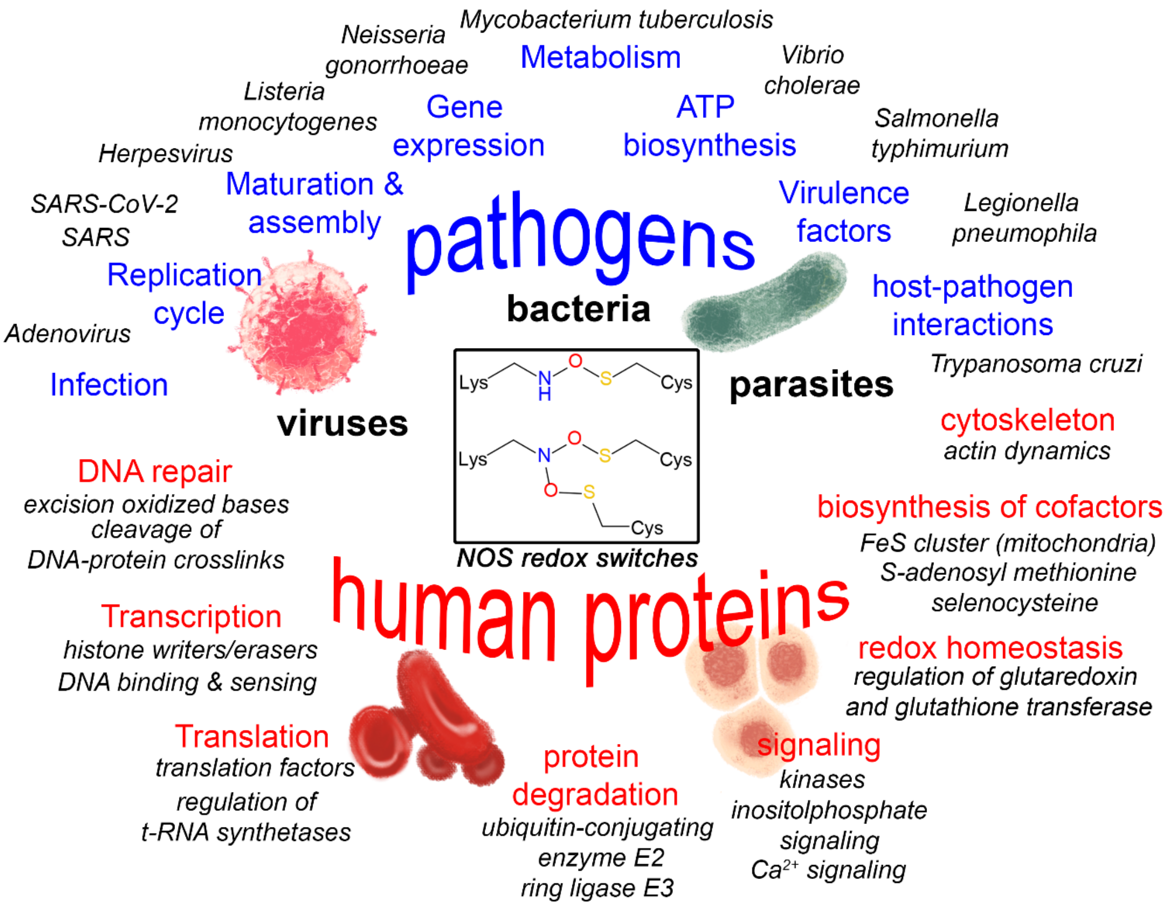

Supplementary Fig. 7. Occurrence and biological functions of proteins containing NOS and SONOS redox switches. Functions of proteins originating from human and plant pathogen are highlighted in blue color, representative examples of relevant species are shown alongside. Cellular functions of human proteins are highlighted in red color, functional subclasses and representative protein families are listed below. Specific information about all proteins regarding origin, biological function, type of NOS/SONOS redox switch, suggested mechanism of the redox switch and potential relevance in disease states is compiled in Supplementary Tables 2 & 3.

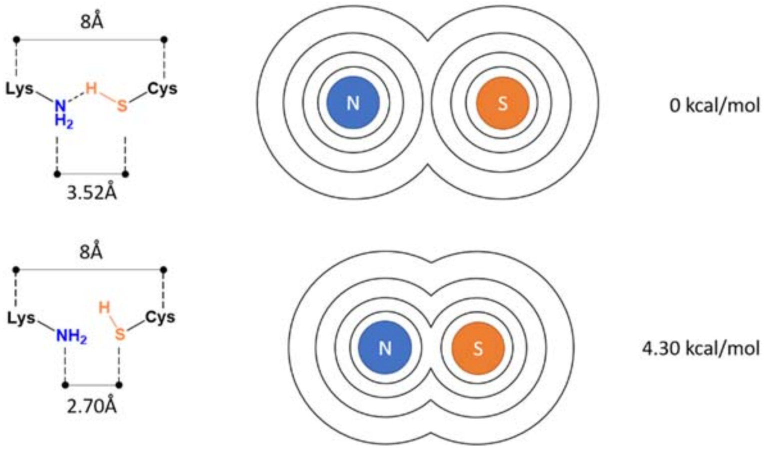

Supplementary Fig. 8. Energy penalty for the constrained N-S-distance of 2.7 Å in NHS (H-bond interaction). Top: electronic energy of the lowest conformer of NHS without the constraint. Bottom: lowest energy conformer of NHS with a constrained N-S distance of 2.7 Å. The electronic energy of the non-constrained conformer is taken as reference.

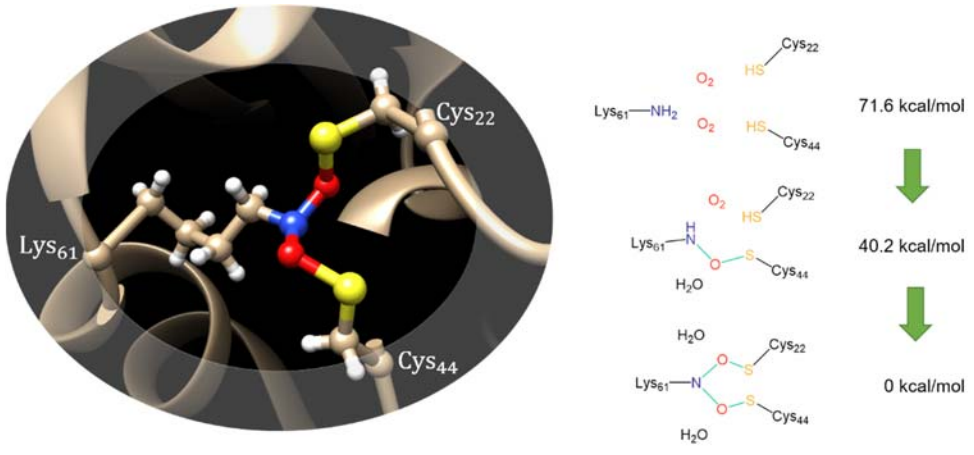

Supplementary Fig. 9. Summary of the thermochemistry in SONOS bond formation using a cluster model of the COVID-19 main protease SONOS site. The calculations were based on the reduced cluster from COVID-19 main protease (PDB code: 7JR4, rebuilt without covalent NOS or SONOS bridge), a structure including only the Cys22, Cys44 and Lys61 residues and truncating the α-carbons (as terminal methyl groups) was taken for the calculation. The starting material contained additionally two oxygen molecules, which were turned into the two NOS linkages and water molecules via two steps. The structures were optimised using B3LYP-D3(BJ)/def2-SVPD to obtain the free energy corrections under standard state conditions (T=298.15 K). The electronic energies were refined using B3LYP-D3(BJ)/def2-TZVPD. Calculations at both levels were carried out with the Gaussian16-A.03 program package under the application of the SMD solvation model (water as solvent). The most thermodynamically stable structure (the SONOS linked cluster) was taken as reference for the energies.

Supplementary Tables

Supplementary Table 1. X-ray crystallographic data collection and refinement statistics.

	*Ng*TAL E93Q	*Ng*TAL wt 0.27 MGy	*Ng*TAL wt 2.7 MGy	*Ng*TAL wt 5.4 MGy
	7OEY	7ODO	7ODP	7ODQ
**Data collection**				
Space group	C 1 2 1	P 21 21 21	P 21 21 21	P 21 21 21
Cell dimensions				
a,b,c (Å)	172.4 55.04 84.37	42.14 82.78 89.36	42.17 82.82 89.4	42.2 82.85 89.42
α, β, γ (°)	90 108.7 90	90 90 90	90 90 90	90 90 90
Resolution (Å)	38.44 - 1.35 (1.398 - 1.35)*	37.55 - 1.40 (1.45 - 1.40)	38.14 - 1.40 (1.45 - 1.40)	37.6 - 1.40 (1.45 - 1.40)
R_{meas}	0.065 (0.865)	0.090 (1.13)	0.099(1.572)	0.112 (2.416)
$CC_{1/2}$	0.998 (0.856)	0.999 (0.856)	0.999 (0.75)	0.999 (0.568)
I/σI	12.67 (1.98)	17.92 (2.41)	16.10 (1.72)	13.85 (1.08)
Completeness (%)	97.80 (97.94)	99.16 (98.07)	99.11 (97.97)	99.08 (97.93)
Multiplicity	4.6 (4.7)	13.4 (13.4)	13.4 (13.4)	13.5 (13.4)
Wilson-B (Å^2)	16.0	13.5	14.2	15.1
**Refinement**				
Reflections used in refinement	160763 (16009)	61847 (6047)	61901 (6040)	61970 (6042)
R_{work}/R_{free}	13.7/16.2	13.9/16.2	14.2/16.6	14.8/17.2
Number of non-hydrogen atoms				
macromolecules	5845	2936	2936	2936
ligands	35	13	13	13
solvent	982	434	434	434
Average B-factor (Å^2)				
macromolecules	22.11	17.43	18.91	20.61
ligands	39.37	22.08	24.75	28.94
solvent	35.06	29.25	31.63	33.85
R.m.s deviations				
RMS(bonds)	0.012	0.006	0.006	0.007
RMS(angles)	1.19	0.93	0.97	1.01

* Values in parentheses are for the highest-resolution shell.

Organism/species	Protein	Function	Redox switch	Mechanism	Disease/comments
**Human Pathogens**					
Viruses					
SARS-CoV-2	Main protease (Mpro)	Polyprotein processing	NOS (K61-C22) **pdb 6XMK** SONOS (C44-K61-C22) **pdb 7JR4**	allosteric mobile Cys (C44)	COVID-19
SARS-CoV	Main protease (Mpro)	Polyprotein processing	NOS (K61-C22) **pdb 3SND**	allosteric	SARS
Human adenovirus	Fiber protein	Binding to host receptor	NOS (K295-C333) **pdb 1UXB**	allosteric	Adenoviral keratoconjunctivitis
Human cytomegalovirus	Nuclear egress complex pUL50	Virus maturation and assembly	NOS (K132-C54) **pdb 6T3X**	allosteric	HCMV infection (herpes)
Bacteria					
Neisseria gonorrhoeae	transaldolase	Sugar metabolism	NOS (K8-C38) **pdb 6ZX4**	allosteric	Gonorrhoea
Neisseria meningitidis	transaldolase	Sugar metabolism	NOS (K8-C38) Sequence homology	allosteric	Bacterial meningitis and septicemia
Vibrio cholerae	Oxaloacetate decarboxylase	Na^+ pump/ATP biosynthesis	NOS (K178-C148) **pdb 2NX9**	Catalytic Lys (CO_2 transfer)	Cholera
Staphylococcus aureus	Pyruvate carboxylase	Gluconeogenesis	NOS (K741-C705) Structural homology **pdb 3HO8**	Catalytic Lys (CO_2 transfer)	Bacterial superinfection
Listeria monocytogenes	Pyruvate carboxylase	Gluconeogenesis	NOS (K710-C674) Structural homology **pdb 4QSK**	Catalytic Lys (CO_2 transfer)	Listeriosis
Listeria monocytogenes	Transcriptional regulator PrfA	Regulation gene expression	NOS (K163-C205) **pdb 6EUT**	allosteric	Listeriosis
Mycobacterium tuberculosis	DAHP synthase (DAHPS)	Amino acid metabolism	NOS (K133-C440) **pdb 3RZI**	substrate binding	Tuberculosis
Pseudomonas aeruginosa	DAHP synthase (DAHPS)	Amino acid metabolism	NOS (K115-C423) Structural homology **pdb 5UXN**	substrate binding	Pneumonia
Ruminococcus gnavus	Metal binding protein	unknown	NOS (K128-C100) **pdb 3U7Z**	allosteric	Crohn's disease
Pseudomonas aeruginosa	Dihydrodipicolinate synthase (putative)	Lysine biosynthesis	NOS (K185-C227) **pdb 3NA8**	catalytic Lys (Schiff base)	Pneumonia
Acinetobacter sp. DL-28	L-ribose isomerase	Sugar metabolism	NOS (K93-C91) **pdb 4Q0P**	substrate binding (Lys)	Nosocomial infections, pneumonia
Salmonella typhimurium	Multidrug resistance regulator RamR	Regulation gene expression	NOS (K63-C67) **pdb 6IE9**	allosteric	Gastroenteritis, typhoid fever
Legionella pneumophila	Effector MavC	Inactivation of human Ub system	NOS (K320-C314) **pdb 6ULH**	Proximal to trans-glutaminase site	Legionnaires' disease
Streptomyces sp. K15	DD-transpeptidase (penicillin binding protein)	Cell wall biosynthesis	NOS (K38-C98) **pdb 1SKF**	catalytic Lys (acid-base)	target protein of β-lactam antibiotics
Parasites					
Trypanosoma cruzi	Farnesyl diphosphate synthase	Isoprenoid biosynthesis	NOS (K158-C154) **pdb 6SDP**	allosteric	Chagas disease
Trypanosoma brucei	Ornithine decarboxylase	Polyamine biosynthesis	NOS (K69-C360) Structural homology **pdb 1F3T**	Catalytic Lys Schiff-base PLP	African sleeping sickness
**Plant pathogens**					
Viruses					
Paramecium bursaria Chlorella Virus	Arginine decarboxylase	Polyamine biosynthesis	NOS (K48-C324) **pdb 2NV9**	Catalytic Lys Schiff-base PLP	Infection of algae, cell lysis and death
Bacteria					
Xanthomonas campestris	Sucrose hydrolase	Sugar metabolism	NOS (K321-C174) **pdb 2WPG**	Proximal to active site	"Black rot" in cruciferous vegetables
Xanthomonas axonopodis	Sucrose hydrolase	Sugar metabolism	NOS (K320-C173) **pdb 3CZG**	Proximal to active site	Bacterial pustule of soybean

Supplementary Table 2. NOS/SONOS bridges in proteins from human and plant pathogens. Information is provided for the protein identity, the origin of the protein, the biological function of the protein, the detected switch type (NOS or SONOS) with residues involved and relevant pdb codes, the suggested mechanism of the redox switch and relevant diseases associated with the identified species. Proteins identified based on structural homology are highlighted in gray shading. The complete list of all proteins with detected NOS bridges is provided in Supplementary Data 1.

Organism	Protein	Function	Redox switch	Mechanism	Disease/comments
DNA repair					
Homo sapiens	OGG1	DNA repair (excision 8-oxo-dG)	NOS (K249-C253) **pdb 1M3Q**	Catalytic Lys (Schiff-base)	Various cancers
Homo sapiens	Metalloprotease Spartan	DNA repair (cleavage of DNA-protein crosslinks)	NOS (K184-C75) **pdb 6MDW**	Proximal to active site	Ruijs–Aalfs syndrome (premature aging and cancer)
Protein biosynthesis and degradation					
Homo sapiens	Density regulated protein	Regulation of translation on ribosome	NOS (K125-C154) **pdb 6VPQ**	Lys on functional loop ('basic loop')	Asperger syndrome<br>Cancer (conjunction with MCTS1)
Homo sapiens	Lysine methyltransferase METTL21C	Methylation of alanine tRNA synthetase	NOS (K76-C160) **pdb 4MTL**	allosteric	Chromosome 4q21 Deletion Syndrome<br>Inclusion body myositis<br>Osteoporosis
Homo sapiens	Ubiquitin-conjugating enzyme E2-25kDa	ubiquitin-dependent protein degradation	NOS (K97-C92) **pdb 3E46**	Catalytic Cys (Catalytic Lys)	Alzheimer's disease<br>Huntington disease
Homo sapiens	Ubiquitin-conjugating enzyme E2 S	ubiquitin-dependent protein degradation (mitosis, meiosis)	NOS (K100-C95) **pdb 6QHK**	Catalytic Cys (Catalytic Lys)	Various cancers
Homo sapiens	Ubiquitin-conjugating enzyme E2 D2<br>E3 ligase RNF38	ubiquitin-dependent protein degradation	NOS ($K8^{E2}$-$C418^{E3}$) Interchain **pdb 4V3L**	Proximal to Zn^{2+} cluster of E3	Various cancers
Transcription regulation					
Mus musculus (conserved in human)	Tubby protein	Transcription regulation, signaling	NOS (K339-C370) **pdb 1I7E**	allosteric	Maturity-onset obesity<br>Retinal dystrophy
Mus musculus (conserved in human)	Homeobox protein Hox-A9	Transcription regulation (cell fate & embryonic patterning)	NOS (K207-C210) **pdb 1PUF**	DNA Binding (Lys)	Acute myeloid leukemia
Homo sapiens	PHD finger protein 2	Transcription regulation<br>Histone demethylation	NOS (K266-C240) **pdb 3PU8**	Substrate binding (Lys) (α-ketoglutarate)	Culler-Jones syndrome, cancer<br>Autism Spectrum Disorder
Homo sapiens	Histone-lysine N-methyltransferase SUV420H2	Transcription regulation<br>Histone methylation	NOS (K122-C111) **pdb 3RQ4**	Proximal to substrate binding site (SAM)	Various cancers
Mus musculus (conserved in humans)	Interferon gamma-inducible protein 16	dsDNA sensing, innate immune response	NOS (K267-C390) **pdb 5YZP**	allosteric	Autoimmune disorders as e.g. primary Sjögren's syndrome (pSS), Cancer
Signaling					
Rattus norvegicus (conserved in humans)	Galectin-1	Signaling, regulation of cell growth and differentiation, immune response	SONOS (C17-K100-C89) **pdb 4GA9**	allosteric	Various cancers, inflammation, allergies
Homo sapiens	Hematopoietic cell receptor CD69	Lymphoid activation, signaling	NOS (K146-C173) **pdb 1E8I**	unknown	Inflammatory diseases (e.g. inflammatory bowel disease)
Homo sapiens	Tyrosine-protein kinase JAK2, pseudokinase domain	Signaling, tyrosine phosphorylation	NOS (K650-C747) **pdb 4FVQ**	allosteric	Bone marrow diseases (Myeloproliferative neoplasms, leukemia)
Gallus gallus (conserved in humans)	Focal adhesion kinase 1	Signaling, regulation cell migration and motility	NOS (K255-C257) **pdb 6CBO**	allosteric	Various cancers
Homo sapiens	Casein kinase I isoform delta	Ser/Thr kinase<br>Signaling in cell division, apoptosis, inflammation	NOS (K57-C71) **pdb 6F1W**	Proximal to ATP binding site	Various cancers<br>Alzheimer's disease<br>Parkinson's disease
Homo sapiens	Dual specificity protein kinase CLK1	Regulation RNA splicing	NOS (K174-C151) **pdb 6FT9**	allosteric	Various cancers<br>Alzheimer's disease
Homo sapiens	Casein kinase gamma 3	Signaling (Ser/Thr kinase)	NOS (K48-C51) **pdb 2IZU**	allosteric	Various cancers<br>Fibrosis
Homo sapiens	TBC1 domain family member 7	Signaling (GTPase activation), regulation cell growth & differentiation	NOS (K233-C286) **pdb 3QWL**	Ligand binding (metal ion)	Macrocephaly/mega-lencephaly syndrome<br>Tuberous sclerosis
Homo sapiens	Leucine carboxylmethyl-transferase-1	Signaling, Regulation PP2A activity	NOS (K62-C36) **pdb 3IEI**	Proximal to substrate SAM	Alzheimer's disease
Rattus norvegicus (conserved in humans)	Rabphilin-3A	Regulation of exo- and endocytosis (neurotransmission)	NOS (K423-C473) **pdb 4NP9**	Effector IP3 binding (Lys) proximal to Ca^{2+} binding site	Levodopa-induced dyskinesias, Martsolf Syndrome (MARTS)
Doryteuthis pealeii	Calexcitin	Signaling in learning and memory, regulation K^+ channels	NOS (K41-C24) **pdb 2CCM**	proximal to Ca^{2+} binding site	Alzheimer's disease
Homo sapiens	diphosphoinositol phosphohydrolase 1	Regulation of inositol diphosphate signaling	NOS (K74-C68) **pdb 6PCK**	allosteric	Renal dysplasia, cerebellar hypoblasia
Biosynthesis sulphur-containing cofactors and rare amino acids					
Homo sapiens	iron-sulfur cluster assembly enzyme ISCU2	Iron-sulfur cluster biogenesis	NOS (K135-C95) **pdb 6UXE**	Catalytic Cys (C95)	Mitochondrial myopathy<br>Friedreich's ataxia (FRDA)<br>Sideroblastic anemia
Homo sapiens	Cysteine desulfurase NFS1<br>iron-sulfur cluster assembly enzyme ISCU2	Iron-sulfur cluster biogenesis	SONOS (C381-K135-C95) Interchain **pdb 6WI2**	Catalytic Cys (C381, C95)<br>Mobile Cys (C381)	Mitochondrial myopathy<br>Friedreich's ataxia (FRDA)<br>Sideroblastic anemia
Homo sapiens	S-adenosylmethionine synthase isoform type-2	S-Adenosylmethionine biosynthesis	NOS (K307-C149) **pdb 6FBP**	allosteric	Familial Thoracic Aortic Aneurysm and Aortic Dissection (FAA)<br>Various cancers
Homo sapiens	Selenophosphate synthetase 1	Redox homeostasis, selenium salvage pathway	NOS (K27-C31) **pdb 3FD5**	On catalytic loop	Ivic Syndrome<br>Ischiocoxopodopatellar Syndrome
Homo sapiens	Selenophosphate synthetase 2	Selenocysteine/selenoprotein biosynthesis	NOS (K58-C62) sequence homology	On catalytic loop	Keshan Disease<br>Ischiocoxopodopatellar Syndrome
Cytoskeleton					
Gallus gallus (conserved in human)	Actin capping protein alpha 1	Regulation of actin filament dynamics	NOS (K135-C147) **pdb 3AA1**	allosteric	Degenerative diseases<br>cancer
Mus musculus (conserved in human)	Profilin-2	Microfilament nucleation and polymerisation	NOS (K126-C16) **pdb 2V8F**	Proximal to ligand binding site	Neurological and behavioural diseases

Supplementary Table 3. Human proteins (or from animal models) with NOS bridges classified according to their cellular function. Information is provided for the protein identity, the origin of the protein, the

biological function of the protein, the detected switch type (NOS or SONOS) with residues involved and relevant pdb codes, the suggested mechanism of the redox switch and relevant diseases associated with the identified species. Proteins identified based on sequence homology are highlighted in gray shading. In case the identified protein originates from an animal model system, a putative sequence conservation of the NOS residues in the human orthologue is indicated. The complete list of all proteins with detected NOS bridges is provided in Supplementary Data 1.

Supplementary Table 4. Steady-state kinetic analysis of *Ng*TAL wild-type and variant Glu93Gln under oxidizing (w/o DTT) and reducing (w/ 1 mM DTT) conditions.

Transaldolase		k_{cat} [s^{-1}]	K_M [mM]	k_{cat}/K_M [s^{-1} mM^{-1}]	DTT
N. gonorrhoeae					
wt ox	steady-state	0.63 ± 0.02	9.98 ± 1.28	0.06	-
	basal	(0.29 ± 0.01) [a]	(9.36 ± 1.33) [a]	(0.03)	
wt red	steady-state	17.63 ± 0.33	5.62 ± 0.41	3.14	+
		(30-fold ↗)	*(2-fold ↘)*	*(60-fold ↗)*	
Glu93Gln ox	steady-state	0.91 ± 0.03	15.12 ± 1.58	0.06	-
	basal	(0.09 ± 0.01) [a]	(6.89 ± 2.06) [a]	(0.01)	
Glu93Gln red	steady-state	15.34 ± 0.24	10.79 ± 0.61	1.42	+
		(20-fold ↗)	*(1.5-fold ↘)*	*(25-fold ↗)*	

Oxidizing conditions, without DTT; reducing conditions, with 1 mM DTT. We estimated k_{cat}, apparent K_M for the substrate D-fructose 6-phosphate (F6P) and catalytic efficiency (k_{cat}/K_M) in a continuous spectrophotometric assay for the conversion of F6P + E4P $\rightarrow$ S7P + G3P as detailed in Methods section. In case of the oxidized, we observed a pronounced lag phase that suggested a catalytic activation under turnover conditions. We thus provide both the steady-state activities after full activation as well as basal activities at t = 0 before activation sets in. The x-*fold* change for k_{cat} and K_M for the reduced enzyme relative to the oxidized form at steady state is indicated. All measurements were carried out in triplicate and are shown as mean ± s.d.

[a] In case of catalytic activation, progress curves were fitted with eq 1 as detailed in the Methods section.
